# Systematic identification of conditionally folded intrinsically disordered regions by AlphaFold2

**DOI:** 10.1073/pnas.2304302120

**Published:** 2023-10-25

**Authors:** T. Reid Alderson, Iva Pritišanac, Đesika Kolarić, Alan M. Moses, Julie D. Forman-Kay

**Affiliations:** ^a^Department of Biochemistry, University of Toronto, Toronto, ON M5S 1A8, Canada; ^b^Department of Molecular Genetics, University of Toronto, Toronto, ON M5S 1A8, Canada; ^c^Department of Cell and Systems Biology, University of Toronto, Toronto, ON M5S 35G, Canada; ^d^Molecular Medicine Program, The Hospital for Sick Children, Toronto, ON M5G 0A4, Canada; ^e^Department of Molecular Biology and Biochemistry, Gottfried Schatz Research Center for Cell Signaling, Metabolism and Aging, Medical University of Graz, Graz 8010, Austria

**Keywords:** AlphaFold2, intrinsically disordered proteins, structural biology, conditional folding, NMR spectroscopy

## Abstract

The AlphaFold Protein Structure Database contains predicted structures for millions of proteins. For the majority of human proteins that contain intrinsically disordered regions (IDRs), which do not adopt a stable structure, it is generally assumed that these regions have low AlphaFold2 confidence scores that reflect low-confidence structural predictions. Here, we show that AlphaFold2 assigns confident structures to nearly 15% of human IDRs. By comparison to experimental NMR data for a subset of IDRs that are known to conditionally fold (i.e., upon binding or under other specific conditions), we find that AlphaFold2 often predicts the structure of the conditionally folded state. Based on databases of IDRs that are known to conditionally fold, we estimate that AlphaFold2 can identify conditionally folding IDRs at a precision as high as 88% at a 10% false positive rate, which is remarkable considering that conditionally folded IDR structures were minimally represented in its training data. We find that human disease mutations are nearly fivefold enriched in conditionally folded IDRs over IDRs in general and that up to 80% of IDRs in prokaryotes are predicted to conditionally fold, compared to less than 20% of eukaryotic IDRs. These results indicate that a large majority of IDRs in the proteomes of human and other eukaryotes function in the absence of conditional folding, but the regions that do acquire folds are more sensitive to mutations. We emphasize that the AlphaFold2 predictions do not reveal functionally relevant structural plasticity within IDRs and cannot offer realistic ensemble representations of conditionally folded IDRs.

## Significance

AlphaFold2 and other machine learning-based methods can accurately predict the structures of most proteins. However, nearly two-thirds of human proteins contain segments that are highly flexible and do not autonomously fold, otherwise known as intrinsically disordered regions (IDRs). In general, IDRs interconvert rapidly between a large number of different conformations, posing a significant problem for protein structure prediction methods that define one or a small number of stable conformations. Here, we found that AlphaFold2 can readily identify structures for a subset of IDRs that fold under certain conditions (conditional folding). We leverage AlphaFold2’s predictions of conditionally folded IDRs to quantify the extent of conditional folding across the tree of life, and to rationalize disease-causing mutations in IDRs.

The accurate prediction of protein structures from amino-acid sequences has been a long-term goal in biology (1, 2). Two deep learning-based methods, AlphaFold2 (3) and RoseTTAFold (4), have recently enabled protein structure prediction with high accuracy (5). DeepMind subsequently predicted the structures for 98.5% of proteins in the human proteome (6). Proteome-wide structural predictions from many organisms are publicly available, in collaboration with the European Bioinformatics Institute, via the AlphaFold Protein Structure Database (AFDB) (https://alphafold.ebi.ac.uk/) (7). Access to high-quality structural predictions has paved the way for a multitude of applications in structural biology (8, 9).

An unexpected effect of the AFDB is that it visually demonstrates the prevalence of intrinsically disordered regions (IDRs). IDRs are predicted to comprise ca. 30% of the human proteome; play important cellular roles as interaction hubs in transcription, translation, and signaling (10, 11); and are enriched in proteins associated with neurological and other diseases (12). Moreover, it has recently become evident that IDRs contribute to and modulate the formation of many in vivo biomolecular condensates via multivalent interactions that lead to phase separation (13, 14). Numerous disease-associated mutations are found in IDRs (15, 16), including mutations implicated in autism spectrum disorder (ASD) and cancer (17), and aberrant phase separation involving IDRs has been linked to diseases such as amyotrophic lateral sclerosis, ASD, and cancer (17, 18), highlighting the need to understand the structural and biophysical impact of these mutations.

At the structural level, IDRs are defined by a lack of stable secondary and tertiary structures and rapid interconversion between different conformations (19, 20). Because of their rapid dynamics, IDRs are not amenable to high-resolution structure determination methods and are frequently removed or not observed in structures determined by X-ray crystallography and cryoelectron microscopy. By contrast, AlphaFold2-generated structural models contain the entire protein sequence, including IDRs (21), and one can now visualize predictions for the significant fraction of the proteome that was previously “dark” and unobservable (22). In addition, AlphaFold2 performs as a state-of-the-art disorder predictor due to a strong correlation between low-confidence AlphaFold2 structural predictions and intrinsic disorder (6, 8).

IDRs, however, do not adopt the static structures that are depicted in the AFDB (21). Instead, IDRs populate an ensemble of interconverting conformations that depends strongly on the primary structure (23–25), and the properties of these ensembles directly have an impact on the functions of IDRs (26–31). However, experimentally determined structural information for IDR conformational ensembles constitutes only a tiny fraction of that available for folded proteins (32, 33), and such ensembles are not deposited in the Protein Data Bank (PDB) (34), which stores the high-resolution structures that were mined to train AlphaFold2 (3) and RoseTTAFold (4). The presence of folded IDR structures (35) in the PDB skews the view of other functional states of IDRs and provides no information for myriad other IDRs that do not fit the “folding-upon-binding” paradigm (36–38).

NMR spectroscopy is well suited to an ensemble-based structural characterization of IDRs at atomic resolution. Indeed, a battery of NMR experiments has been applied to probe the conformations of IDRs and residual structure therein (39–42), with dedicated software programs focused on integrating NMR and other biophysical methods to determine ensemble representations of IDRs that best agree with the experimental data (43–46). However, both the integrative structural biology approach used to determine ensemble representations of IDRs and the NMR-driven determination of residual structure or secondary structure propensity are not deposited in the PDB, which is used to train and validate deep-learning models. Finally, because AlphaFold2 was trained on a subset of the PDB that excluded NMR structures (3), NMR data offer a unique validation metric to assess the accuracy of predicted AlphaFold2 structures in solution, as recently demonstrated (47–49).

Here, we show that thousands of IDRs are predicted by AlphaFold2 to be folded with high (70 ≤ x < 90) or very high (≥90) predicted local difference distance test (pLDDT) scores, which measure the confidence in the predicted structures. We find that, compared to IDRs with low pLDDT scores, the amino-acid sequences of IDRs with high pLDDT scores show more positional conservation. Only 4% of IDR sequences with high pLDDT scores have alignment matches in the PDB, indicating that structural templating is not the reason that AlphaFold2 confidently folds these IDRs. For a subset of IDRs that fold under specific conditions, such as in the presence of binding partners (35) or following post-translational modification (PTM) (50), and have been extensively characterized by NMR spectroscopy, we find that the AlphaFold2 structures of these IDRs resemble the conformation of the folded state. Moreover, for more than 1,400 IDRs that are known to fold under specific conditions, we observed that the AlphaFold2 confidence scores enable the prediction of conditional folding. This suggests that AlphaFold2 can systematically identify disordered regions that fold upon binding or modification. We found that IDRs with high-confidence AlphaFold2 scores are enriched in disease-associated mutations relative to IDRs with low-confidence scores. We leveraged AlphaFold2 to compare conditional folding in eukaryotes, bacteria, and archaea and found that prokaryotes show much higher proportions of conditionally folding IDRs, leading us to conclude that a large majority of eukaryotic IDRs function without adoption of structure. We propose that IDRs with high pLDDT scores may fold in the presence of specific binding partners or following PTMs, which we refer to as conditional folding.

## Results

In this work, we focus on the structural predictions that are available in the AFDB (7), which contains precomputed AlphaFold2 models that can be easily visualized and downloaded for offline inspection. Moreover, for IDRs, we find that the structural predictions of full-length proteins from the versions of AlphaFold2 that have been implemented as Jupyter Notebooks on Google Colaboratory (6, 51) are generally of lower quality and do not agree well with AFDB (*SI Appendix*, Fig. S1). As such, we focus henceforth exclusively on structural predictions within the AFDB. We define a conditionally folded protein as any protein that is disordered in the absence of 1) a binding partner or ligand or 2) PTM, which then can acquire a stable fold in their presence. While there are alternative definitions of conditionally folded proteins (52), we favor our definition because it acknowledges the complex free energy landscapes of these proteins and the subsequent responsiveness to the compositions, localizations, and concentrations of binding partners and the types, sites, and stoichiometries of PTMs.

**Fig. 1.**
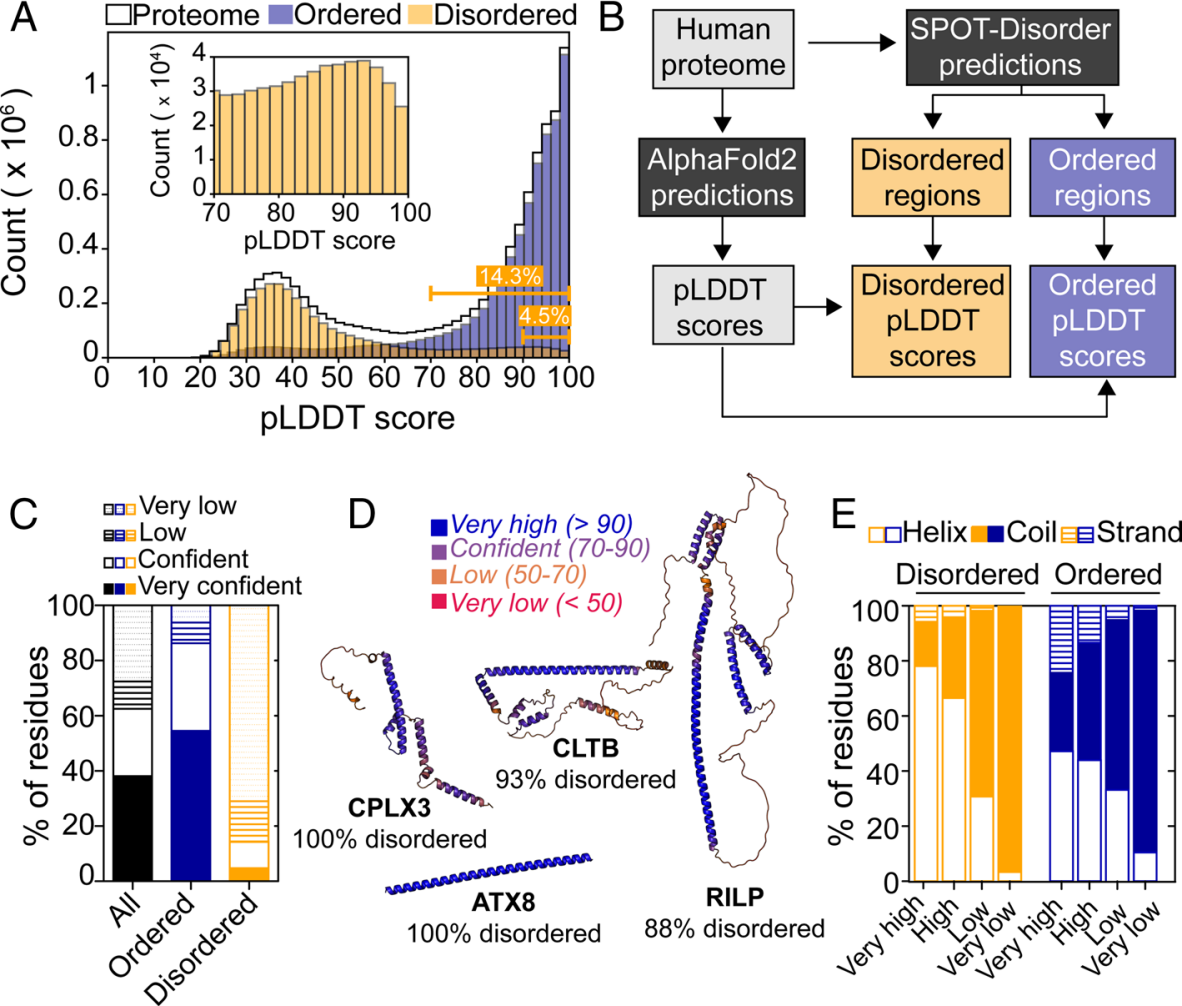
Predicted IDRs in the human proteome that have confident structures in the AFDB. (*A*) Histogram of per-residue pLDDT scores in the human proteome (black) compared with the predicted disordered (orange) or ordered (blue) regions. The *Inset* shows an expansion of the predicted disordered regions between pLDDT scores of 70 to 100. The cumulative percentage of predicted disordered residues with scores greater than or equal to 70 and 90 are indicated in the lower right. (*B*) Flowchart outlining the analysis presented in (*A*). (*C*) Stacked bar graph showing the percentage of residues in the human proteome (black) that have very low (<50; dotted lines), low (50 ≤ *x* < 70; horizontal lines), confident (≤ 70 *x* < 90; empty), and very confident (≤90; filled) pLDDT scores. The corresponding plots are included for SPOT-Disorder-predicted disordered residues (orange) and ordered residues (blue). (*D*) Example structures in the AFDB for SPOT-Disorder-predicted IDRs, with the percentage of predicted disordered residues of the total listed. The AFDB structures have been color-coded by pLDDT scores as indicated (*E*) DSSP-determined secondary structure content of the predicted disordered (orange) and ordered (blue) regions as a function of pLDDT thresholds.

We first analyzed the distribution of per-residue pLDDT scores in the human AFDB (Fig. 1*A*). The histogram of pLDDT scores shows a clear bimodal distribution, with the two local maxima centered around values of 100 and 35 (Fig. 1*A*). The majority of residues, accounting for 62.6% of the proteome, have pLDDT scores greater than 70 (Fig. 1*A*), which is defined to be the lower threshold for a “confident” score. The remaining 37.4% of residues in the proteome have pLDDT scores below 70 (“low”), while 27.8% of residues have scores below 50 (“very low”). Thus, while a significant percentage of residues have confident or “very confident” pLDDT scores, suggesting that the predicted structures of regions are expected to be accurate, there also exists a sizeable fraction of residues that have low to very low pLDDT scores (Fig. 1*A*), indicative of low-accuracy structural regions that should not be interpreted quantitatively.

### Predicted Human IDRs with High pLDDT Scores in the AFDB.

To determine how many IDRs in the AFDB have high pLDDT scores that reflect high confidence structural predictions, we extracted the predicted IDRs from the human AFDB (Fig. 1*B*). We used the state-of-the-art sequence-based predictor of intrinsic disorder, SPOT-Disorder (53), to calculate the predicted disorder propensities for each protein in the human proteome (Fig. 1*B*). A total of ca. 3.5 million residues are predicted to be disordered, totaling 32.8% of the human proteome, consistent with expectations based on previous reports (54) (*SI Appendix*, Table S1). We then investigated the proteins that comprise the lower end of the distribution of pLDDT scores within the AFDB. An inverse correlation between pLDDT scores and predicted disorder was previously noted (6), with pLDDT scores reported to perform well as a predictor of intrinsic disorder (6, 54). Thus, one might assume that the 32.8% of predicted residues in IDRs would be embedded in the 37.4% of residues with pLDDT scores below 70 because IDRs should have low or very low confidence structural predictions.

However, when we isolated the pLDDT scores from residues that localize to SPOT-Disorder predicted IDRs (Fig. 1*B*), we found that IDRs also have a bimodal distribution of pLDDT scores (Fig. 1*A*). Of the ca. 3.5 million predicted disordered residues, 14.3% (i.e., ca. 500,000 residues in total) have confident pLDDT scores greater than or equal to 70 (Fig. 1 *A* and *C* and *SI Appendix*, Table S2). When the pLDDT threshold is increased to greater than or equal to 90 (very confident), there are more than 160,000 residues that remain, accounting for 4.5% of the total number of disordered residues (Fig. 1 *A* and *C*). This analysis indicates that there is a significant fraction of SPOT-Disorder-predicted IDRs in the human AFDB that has high-confidence structural predictions (Fig. 1*A* and *SI Appendix*, Fig. S2), and therefore, the assumption that all IDRs have low pLDDT scores is incorrect.

Because the prevalence of confidently predicted structures within IDRs was unexpected, we sought to ensure that the confident and very confident scores associated with SPOT-Disorder-predicted IDRs are not the result of poor or biased disorder predictions. To this end, we extracted the pLDDT scores for IDRs in the DisProt database of experimentally validated IDRs (55). There is a total of 932 human IDRs in DisProt, yielding over 300,000 residues that can be used as a direct comparison to the SPOT-Disorder predictions. We find that the distribution of pLDDT scores for IDRs in DisProt shows an even higher proportion of residues with confident scores greater than or equal to 70 than the SPOT-Disorder-predicted IDRs: nearly 30% of the experimentally validated DisProt IDRs have confident pLDDT scores (*SI Appendix*, Fig. S2). Thus, the IDRs that were predicted by SPOT-Disorder do not contain an artificially inflated fraction of residues with high pLDDT scores. In addition, we checked whether the high pLDDT scores might originate from a few disordered residues that are immediately adjacent to structured domains. To this end, we filtered the predicted IDRs with confident pLDDT scores (greater than or equal to 70) for those with consecutive regions of disorder. We found that over 50% of IDRs with high pLDDT scores come from stretches of 24 or more consecutive disordered residues, with nearly 10% arising from IDRs that have 100 or more consecutive disordered residues (*SI Appendix*, Fig. S2). Finally, we filtered the list of IDRs to extract those that have 10 and 30 or more consecutive residues with confident (very confident) pLDDT scores. We identified 14,996 (4,883) and 3,730 (1,157) IDRs that respectively match these criteria (*SI Appendix*, Table S3).

From the list of proteins that contain predicted IDRs equal to or longer than 30 residues with high pLDDT scores, we selected a handful of examples for structural analysis in the AFDB (Fig. 1*D*). In particular, we identified proteins that are predicted to be predominantly disordered by SPOT-Disorder yet are assigned very high pLDDT scores in the AFDB. For example, the protein ataxin-8 (UniProt ID: Q156A1) contains an initial Met residue followed by 79 Gln residues and is predicted to be fully disordered (Fig. 1*D*). However, the AlphaFold2 model indicates that ataxin-8 forms a single α-helical structure with pLDDT scores greater than 90 for every residue in the helix (Fig. 1*D*). Similarly, the proteins complexin-3 (UniProt ID: Q8WVH0), clathrin light chain B (UniProt ID: P09497), and Rab-interacting lysosomal protein (RILP, UniProt ID: Q96NA2) all adopt highly α-helical structures with very high pLDDT scores and various degrees of tertiary interactions (Fig. 1*D*), despite being predicted by SPOT-Disorder to be almost entirely disordered (88 to 100% predicted disorder).

Given that the above examples were α-helical structures, we computed the secondary structure content for every model in the AFDB to assess the structural properties of IDRs with high-confidence pLDDT scores. This analysis revealed primarily helical conformations in the high and very high confidence IDR structures (Fig. 1*E*). When compared to ordered regions, the predicted IDRs are significantly enriched in helical conformations at the expense of coils and strands (Fig. 1*E* and *SI Appendix*, Table S4). In addition, we note that the predicted IDRs with low confidence scores still exhibit significant secondary structure content: over 32% of residues with pLDDT scores between 50 and 70 are assigned to regions of the secondary structure as compared to 38% in ordered regions (Fig. 1*E* and *SI Appendix*, Table S4). In the IDRs with pLDDT scores below 50, the percentage of residues in regions of secondary structure dramatically diminishes to only 3.4% (Fig. 1*E* and *SI Appendix*, Table S4).

Overall, the analysis of secondary structure content in predicted IDRs with high pLDDT scores shows an enrichment in helical conformations. Moreover, the selected examples in Fig. 1*D* all have at least one long, extended α-helix that is not stabilized by tertiary contacts. These so-called single α-helix (SAH) domains are well known in the literature and are estimated to exist in 0.2 to 1.5% of human proteins (56, 57), with the formation of SAHs dependent on stabilizing *i* to *i*+4 salt bridges between charged side chains (58). Thus, SAH-like structures in the AFDB for sequences predicted to be IDRs may be plausible and physically reasonable, perhaps including a combination of SAHs and long α-helices that form stabilizing intermolecular contacts (e.g., coiled coils).

### Comparing NMR Data and AlphaFold2 Structures for Experimentally Characterized IDRs.

**Fig. 2.**
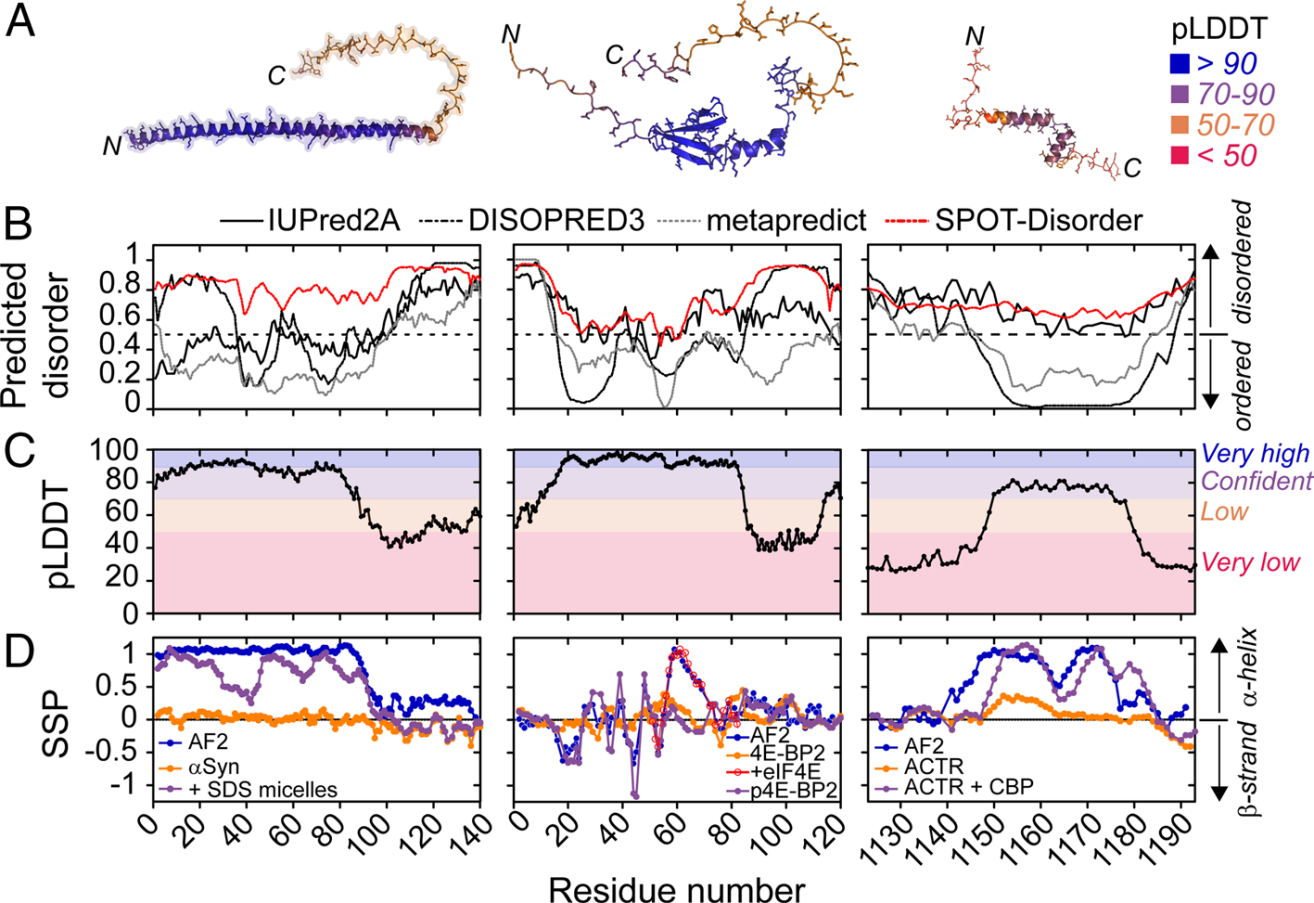
Examples of three IDRs with high pLDDT scores that conditionally fold and have been characterized by NMR spectroscopy. (*A*) AlphaFold2-predicted structures of three IDPs/IDRs that are color coded by pLDDT scores. From left to right: α-synuclein, 4E-BP2, and ACTR. The N and C termini of each protein are indicated. (*B*) Sequence-based prediction of disorder for the three IDPs/IDRs. Four different programs were used: IUPred2A, DISOPRED3, metapredict, and SPOT-Disorder. Only SPOT-Disorder correctly predicts the disordered nature of all three IDPs/IDRs. (*C*) Per-residue pLDDT confidence scores derived from the AlphaFold2 structures. (*D*) NMR ^13^Cα and ^13^Cβ chemical shift-derived secondary structure propensity (SSP) (67). For the AlphaFold2 structures (blue) and the 4E-BP2 peptide bound to eIF4E (PDB ID: 3am7), NMR chemical shifts were back-calculated from the structure using SPARTA+ software (68). The unbound/unmodified IDPs/IDRs (orange) show very little preferential secondary structure (α-synuclein) or modest populations of helix (4E-BP2, ACTR). By contrast, the binding to SDS micelles (α-synuclein), phosphorylation (4E-BP2), binding to eIF4E (4E-BP2), or the binding to CBP (ACTR) induces the formation of stable secondary structure (purple) that is in better agreement with the AlphaFold2 structures.

Given that our structural analyses above relied on sequence-based predictions of intrinsic disorder, we asked whether the structures of IDRs with high pLDDT scores show correspondence with experimentally determined structural propensities of IDRs. To this end, we focus on three IDRs/IDPs that have been characterized in detail by NMR spectroscopy (50, 59–63). Two of the model proteins, α-synuclein (UniProt ID: P37840) and 4E-BP2 (UniProt ID: Q13542), are full-length IDPs, whereas the third protein, ACTR or NCoA3 (UniProt ID: Q9Y6Q9), is a small IDR that is part of a larger protein with folded domains and other longer IDRs. The AlphaFold2-predicted structures of the three proteins (Fig. 2*A*) vary from all helical (α-synuclein, ACTR) to a mixture of strand and helix (4E-BP2). For each structure, the pLDDT scores in the regions of the secondary structure range from high to very high (Fig. 2*B*), suggestive of atomic-level accuracy and an overall high level of confidence in the structural models (3, 7). Next, we checked the predicted disorder propensity using four different sequence-based predictors of intrinsic disorder (53, 64–66), and we found that either two (4E-BP2, ACTR) or three (α-synuclein) of the four programs predicted that these proteins would be predominantly ordered (Fig. 2*C*). Thus, without additional experimental evidence, an AFDB user who relies on the overlap between sequence-based disorder prediction software and the (confident) AFDB structure would likely assume that the IDR/IDP under investigation folds into the high-confidence predicted structure.

However, we find that there is disagreement between experimental NMR data from these IDRs/IDPs and the AlphaFold2 models and sequence-based prediction of disorder (Fig. 2*D*). It is well known that ^13^Cα and ^13^Cβ chemical shifts are sensitive reporters on the secondary structure of a protein (69). For each residue in the protein, the expected chemical shifts for a fully disordered state can be subtracted from the measured chemical shifts. These so-called secondary chemical shifts (corrected for neighboring residues) provide residue-level information regarding the secondary structure of a protein, including the fluctuating, fractional secondary structure of disordered regions, which can be quantified using software programs such as SSP, CheSPI and δ2D (67, 70, 71). If the AFDB structures were correct, the expected secondary chemical shifts would reveal long stretches of secondary structure with “fractional” structure values near 1 or −1 for a fully stabilized α-helix or a β-strand, respectively (Fig. 2*D*). By contrast, the experimental NMR data for each of three proteins in Fig. 2 show that there is no stable secondary structure and only a fractional preference to populate secondary structure (Fig. 2*D*). Thus, a user without knowledge of the disordered nature of these proteins from experiment could erroneously trust the confident AlphaFold2 models (Fig. 2 *A* and *C*) and use the lack of predicted disorder (Fig. 2*B*) as a cross-validation method to justify the structures.

Interestingly, however, there are correlations between the AFDB structures of these IDRs/IDPs and their experimentally defined conformations under specific conditions. For example, the N terminal ca. 100 residues of α-synuclein fold into a long α-helix in the presence of lipid vesicles (61), and the AFDB structure reflects this lipid-bound conformation (Fig. 2*A*). There is no high-resolution structure of the lipid-bound state, so no side-by-side structural comparison can be made; assigned NMR chemical shifts are only available for α-synuclein bound to SDS micelles (72) (Fig. 2*D*, purple). The solution structure of 4E-BP2 revealed that it folds into a four β-strand structure upon multisite phosphorylation (PDB ID: 2mx4) (50), and the AFDB structure has correctly identified the β-strands and the intermolecular contacts: the heavy-atom RMSD is 0.35 Å upon alignment of the β-strands from T19-D55 in the experimental structure to the AFDB model. Confusingly, however, an additional helix in the AFDB model is present in residues R56 to R62, followed by a short turn and then a 3_10_-helix between residues P66-Q69. These additional helices resemble those seen in crystal structures of fragments of non-phosphorylated 4E-BP2 and 4E-BP1 bound to eukaryotic translation initiation factor 4E (eIF4E) (PDB IDs: 3am7, 5bxv). For ACTR, a helix–turn–helix motif is present in the AFDB structure, whereas a three-helix structure is formed upon binding to CBP (PDB: 1kbh). ACTR and 4E-BP2 provide particularly useful test cases because the experimental structures (PDB ID: 1kbh, 2mx4) were determined by NMR spectroscopy, and AlphaFold2 was not trained on NMR structures (3).

Finally, we note that fractional secondary structure in the unbound form of an IDR does not necessarily correlate with the secondary structure in the AFDB model of the IDR. For example, in the unbound form of ACTR, there is an α-helix populated to approximately 40% between residues 1,150 to 1,163 (Fig. 2*D*, orange), which closely matches the position of the first α-helix in the AFDB (Fig. 2*D*, blue) and experimental structures (Fig. 2*D*, purple). However, the second and third α-helices in the experimental structure are not appreciably formed in the unbound state or the AFDB model (Fig. 2*D*, orange). The lack of clear correlation between fractional secondary structure in an isolated IDR and stabilized structure in complexes has been noted previously (73).

These comparisons show that the high-confidence AFDB structures of IDRs do not reflect the conformational ensemble sampled by the unbound or unmodified form of the IDR. Instead, the AlphaFold2 structures appear to resemble conditionally folded states. Moreover, in the case of 4E-BP2, the AlphaFold2 model has combined structural features from two different conditionally folded forms of the protein that do not coexist: One structure forms upon multisite phosphorylation (β-strand-rich) and the other upon binding to eIF4E (helical). In this case, the AlphaFold2 structure of 4E-BP2 obscures the molecular mechanism of the protein function (see section below).

### AlphaFold2 Structures of Experimentally Characterized IDRs Resemble the Conditionally Folded State but Do Not Capture Structural Plasticity.

**Fig. 3.**
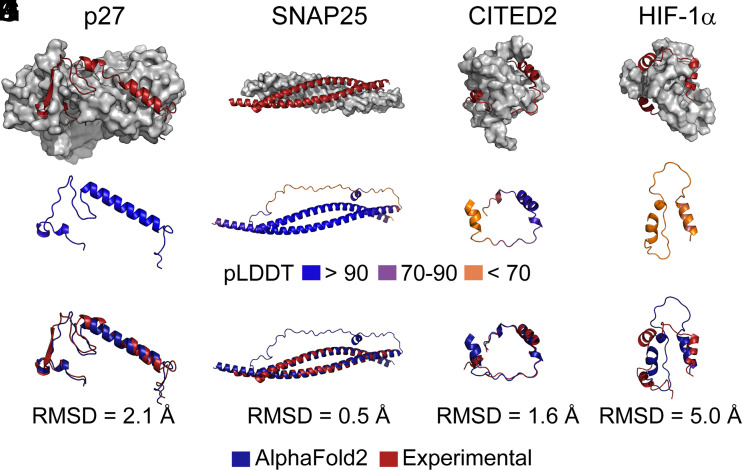
Structures of IDRs in the AFDB correlate with experimentally determined structures of the IDRs bound to interaction partners. (*A–D*) experimental structures for the listed IDRs/IDPs (red) bound to an interacting folded domain (grey surface representation). The PDB ID codes are 1jsu, 1kil, 1p4q, and 1l8c respectively. (*E*–*H*) The predicted structures in the AFDB for the listed IDRs/IDPs in panels *A*–*D*. These structures have been color-coded by pLDDT scores, with blue, purple, and orange respectively corresponding to very confident (≥90), confident (70 to 90), and low (<70) scores. (*I*–*L*) comparison of the experimental structures from panels *A*–*D* with the predicted structures in the AFDB from panels *E*–*H*. Experimental structures are colored red and AFDB structures blue. The heavy-atom RMSD upon alignment of secondary structure elements is indicated.

Our analysis of IDRs/IDPs with extensive NMR data showed that AFDB models with high pLDDT scores might reflect a conformation of the IDR/IDP that only forms under specific conditions. We thus examined the AlphaFold2 structures of an additional four IDRs/IDPs that are known to fold upon binding to interacting partners and have high-resolution structures of the complex in the PDB. The structures for two of these complexes were determined by X-ray crystallography (p27: 1jsu; SNAP25: 1kil) and two were determined by NMR spectroscopy (HIF-1α: 1l8c; CITED2: 1p4q). A comparison of the experimental structures (Fig. 3 *A*–*D*) with those in the AFDB (Fig. 3 *E*–*H*) shows an overall high structural similarity (Fig. 3 *I*–*L*). For the two examples with very confident AFDB structures (p27, SNAP25), the heavy atom RMSDs when comparing the experimental and the AFDB structures are 0.5 and 2.1 Å (Fig. 3 *E*, *F*, *I*, and *J*). Even for some structures that have a mixture of very confident and low pLDDT scores (CITED2, Fig. 3 *F* and *G*), or only low pLDDT scores (HIF-1α, Fig. 3*H*), the overall architecture of the AFDB structure resembles that of the experimental structure, with RMSD values of 1.6 (Fig. 3*K*) and 5.0 Å (Fig. 3*L*), respectively. Taken together, these analyses suggest that the AFDB structures formed by IDRs with high pLDDT scores are likely capturing some structural features that form in the presence of specific interactions. In the case of an IDR with very low pLDDT scores (HIF-1α), the regions of the secondary structure appear to correlate with the bound-state conformation.

If AlphaFold2 structures of IDRs reflect the conditionally folded state, we were interested to determine whether these structures can inform on the molecular mechanisms of IDRs. The interconversion between different structural forms is essential for IDR function, and it is well known that IDRs can bind to multiple interaction partners via different interfaces or motifs, oftentimes forming unique structural elements in the process (35). We selected three IDRs with multiple experimentally determined structures in which the IDR has folded into a different conformation. Some of the experimental structures of these IDPs or IDRs from the cystic fibrosis transmembrane conductance regulator (CFTR; UniProt ID: P13569), SNAP25, and 4E-BP2 show good agreement with the AlphaFold2 models (*SI Appendix*, Fig. S3). However, AlphaFold2 returns only a single structural model of these IDRs (*SI Appendix*, Fig. S3), which as we discuss in *SI Appendix*, *Supplementary Appendix* is not compatible with the known structural plasticity of these IDRs.

### Experimental Assessment of the Accuracy of AlphaFold2 Predictions for IDRs.

Given that the AFDB contains only a single structure of a given IDR, our analyses above emphasize that these AlphaFold2 models are but one possible conformation of the IDR, especially in the context of an IDR binding to an interaction partner. We discuss how NMR and other biophysical data can rapidly assess the accuracy of AlphaFold2 predictions for IDRs (*SI Appendix*, *Supplementary Appendix* and Fig. S4), including NMR data without resonance assignments (*SI Appendix*, Fig. S5). Moreover, if high-confidence structures of IDRs/IDPs are capturing the bound/modified states of IDRs/IDPs, as our results above suggest, then we wondered whether such structures could be used with protein-protein docking software to obtain structural models of IDR/IDP complexes bound to globular domains. We explored the possibility of rigid-body docking an IDR with a high-confidence AlphaFold2 predicted structure in *SI Appendix*, *Supplementary Appendix*. Although we only examined a single case involving the CITED2 transactivation domain binding to the folded CBP TAZ domain (*SI Appendix*, Fig. S6), our results and those of docking studies in the literature suggest that AlphaFold2 models require additional considerations before usage for molecular docking (74).

### Predicted IDRs with High pLDDT Scores Are Enriched in Charged and Hydrophobic Residues.

We next sought to understand why AlphaFold2 is folding some IDRs/IDPs into high-confidence structures. At least three non-mutually exclusive hypotheses could explain the prevalence of high pLDDT scores in predicted IDRs: (i) global amino-acid sequence differences in comparison to the predicted IDRs with low pLDDT scores, (ii) strong signals of co-evolution among residues in these regions, which would imply “high quality” multiple sequence alignments (MSAs) that are unusual for IDRs, and (iii) the enrichment of high-pLDDT IDR sequences in the PDB. The first possibility would reflect a differential “folding propensity” that is inherently encoded in the amino-acid sequences of high vs. low pLDDT-scoring IDRs, whereas the latter two possibilities would influence the AlphaFold2 prediction confidence due to (ii) the depth of the MSAs or (iii) sequence similarity to the structures from the PDB used in training (75). Given the relatively poor coverage of IDRs in the PDB (55) and the poor positional alignability for most IDRs (76–79), it is plausible that some combination of all three of the aforementioned possibilities could contribute to high pLDDT scoring IDRs.

**Fig. 4.**
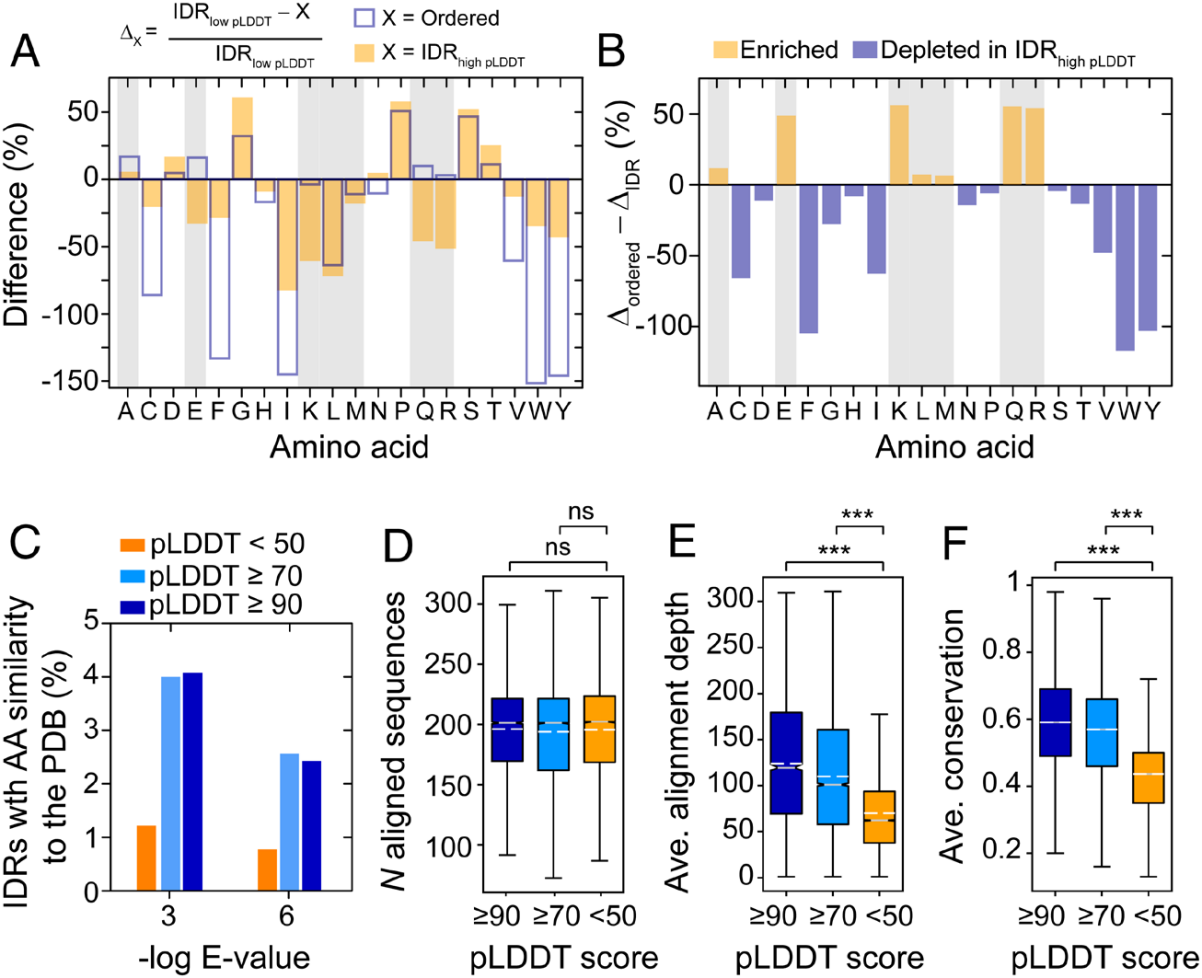
Bioinformatics analysis of predicted IDRs in the AFDB with high pLDDT scores. (*A*) Amino-acid percentages in the regions of predicted order and disorder, with the disordered regions further separated into those with confident pLDDT scores greater than or equal to 70 (IDR_high pLDDT_) and those below 50 (IDR_low pLDDT_). Shown here is the percent change in the relative amino-acid percentages for IDR_low pLDDT_ and either ordered regions (Δ_Order_, empty blue bars) or IDR_high pLDDT_ (Δ_IDR,_ orange bars). Positive values indicate that a given amino acid is fractionally enriched in IDR_low pLDDT_ whereas negative values indicate depletion. (*B*) The difference between Δ_Order_ and Δ_IDR high_ reports on the relative difference in amino-acid usage between ordered regions and IDR_high pLDDT_ regions as compared to IDR_low pLDDT_ regions. Positive values reflect an increased usage of a given amino acid in IDR_high pLDDT_ regions whereas negative values reflect enrichment in ordered regions, as compared to IDR_low pLDDT_ regions. (*C*) BLAST results from querying amino-acid sequences in the PDB (*Methods*) for predicted IDRs in the AFDB that are longer than 10 residues. Percentage of predicted IDRs (hits/total) that were identified in the PDB as a function of the *E*-value and the pLDDT score, with <50 in orange, ≥70 in cyan, and ≥90 in blue. Box plots of the number of aligned sequences (*D*), average alignment depth (*E*), and average positional conservation (*F*). Panel *D* is not significant (*ns*) whereas panels *E* and *F* have *P*-values (Mann-Whitney) < 0.0001 when comparing pLDDT < 50 and the other groups (***).

To gain insight into these possibilities, we first computed the amino-acid frequencies for each of the following three categories: predicted disordered regions with low pLDDT scores below 50 (IDR_low pLDDT_), predicted disordered regions with high pLDDT scores greater than or equal to 70 (IDR_high pLDDT_), and predicted ordered regions (ordered). We hypothesized that the amino-acid frequencies in IDR_low pLDDT_ should reflect the sequence biases found in disordered regions, i.e., an enrichment in some charged (D, E, K), polar (Q, S, T), small (G), and helix-disrupting (P) residues (55). Indeed, the difference between IDR_low pLDDT_ and ordered regions (Δ_ordered_) shows that IDR_low pLDDT_ sequences are enriched in the expected residues that are known to promote disorder (Fig. 4*A* and *SI Appendix*, Fig. S7).

We next compared IDR_low pLDDT_ and IDR_high pLDDT_ regions (Δ_IDR_) to determine whether there are global differences in the sequences of these IDRs that are encoded within per-residue pLDDT scores. Surprisingly, we found that IDR_high pLDDT_ sequences are significantly enriched in E, K, Q, and R residues relative to IDR_low pLDDT_ sequences, as evidenced by the large differences in values of Δ_ordered_ and Δ_IDR_ for these residues (Fig. 4*A*). Furthermore, IDR_high pLDDT_ sequences have fewer disorder-promoting residues (e.g., P, S, T, D, G) and more order-promoting residues (e.g., C, F, I, L, V, W, Y) than IDR_low pLDDT_ sequences. Nonetheless, IDR_high pLDDT_ sequences still resemble IDR sequences when analyzed by mean net charge and mean hydropathy (*SI Appendix*, Fig. S8), which is a sequence metric that identifies IDRs from ordered regions (80). However, the IDR_high pLDDT_ sequences show a much broader distribution in both the mean hydropathy and mean net charge dimensions than the IDR_low pLDDT_ sequences (*SI Appendix*, Fig. S8). Thus, although the IDR_high pLDDT_ sequences contain more disorder-promoting residues than ordered regions (Fig. 4*B*), IDR_high pLDDT_ sequences appear to have a mixture of both order- and disorder-promoting residues.

### IDRs with High pLDDT Scores Have Limited Similarity to PDB Sequences but Are More Positionally Conserved than IDRs with Low pLDDT Scores.

Next, we searched the PDB for IDRs with positional sequence similarity. We hypothesized that there should be more IDR_high pLDDT_ sequences with similarity to sequences in the PDB than IDR_low pLDDT_ sequences. An enrichment in similarity for IDR_high pLDDT_ sequences could indicate that AlphaFold2 is matching template structures of these IDRs that were used in training. Indeed, we found that IDR_high pLDDT_ sequences are significantly enriched over IDR_low pLDDT_ sequences for similarity to PDB sequences (Fig. 4*C*). The percentage of IDRs with pLDDT scores ≥70 that have confident BLASTP hits (E-value < 1e-3 or < 1e-6) in the PDB is more than threefold higher than IDRs with low pLDDT scores (Fig. 4*C*). However, it is important to note that the percentage of high-quality hits in the PDB relative to the total number of predicted IDRs in each pLDDT threshold is very low, i.e., a maximum hit rate of 4% was obtained (Fig. 4*C*). Therefore, AlphaFold2 has not simply templated structures of IDRs from the PDB, as the overall coverage of IDR sequences in the PDB remains below 4%.

We next asked whether the confident structural predictions for IDRs with high pLDDT scores could reflect higher alignment quality as compared to IDRs with low pLDDT scores. IDRs that conditionally fold have been previously shown to have higher levels of positional amino-acid conservation than IDRs in general (76, 81). To compute the positional sequence conservation, we constructed MSAs for predicted IDR sequence across different pLDDT categories using homologous sequences retrieved from the Ensembl database (82). The MSAs contained nearly identical numbers of sequences for each of the three classes of IDRs (pLDDT scores < 50, ≥ 70, and ≥ 90) (Fig. 4*D*), yet the average alignment depth was significantly enriched in IDRs with pLDDT scores ≥ 70 and ≥ 90, relative to those with pLDDT scores < 50 (*P*-value < 0.0001, Mann-Whitney) (Fig. 4*E*). Moreover, the quality of the alignments was higher for IDRs with high pLDDT scores compared to those with low pLDDT scores, as evidenced by greater levels on average of positional conservation (*P*-value < 0.0001, Mann-Whitney) (Fig. 4*F*).

Overall, our sequence analysis of predicted IDRs demonstrates that those with high pLDDT scores have higher sequence similarity to the sequences in the PDB than predicted IDRs with low pLDDT scores. However, the overall coverage of IDR sequences in the PDB remains low, with only 4% of high-scoring IDR sequences displaying similarity (E-value < 0.001) to PDB sequences. More significantly, IDRs with high pLDDT scores are more positionally conserved, with nearly 60% sequence identity on average (ignoring gaps; see *Methods*) and contain fewer gaps than predicted IDRs with low pLDDT scores. Given that AlphaFold2 relies on MSAs as input for its structural predictions (3), these results provide insight into why AlphaFold2 is folding IDRs with high pLDDT scores into confident structures. The fact that IDRs with high pLDDT scores only rarely have sequence homologs in the PDB suggests that the dominant forces behind the AlphaFold2 predictions for these IDRs are high-quality MSAs and the underlying amino-acid compositions (83), and not structural templating.

### AlphaFold2 Confidently Assigns Structures for the Majority of IDRs Known to Conditionally Fold.

Our bioinformatics analyses provided evidence that IDRs with high pLDDT scores have both compositional differences from and higher quality MSAs than IDRs with low pLDDT scores (Fig. 4). IDRs that have high levels of positional conservation are more likely to conditionally fold (76, 81). Given that our structural analyses above were limited to a handful of conditionally folded IDRs (Figs. 2 and 3 and *SI Appendix*, Fig. S3), we sought to gain broader insight into whether the predicted IDRs with high pLDDT scores are, indeed, IDRs that conditionally fold.

To this end, we first investigated the per-residue pLDDT scores for proteins in five databases of conditionally folded IDRs/IDPs across different organisms: Disordered Binding Sites (DIBS) (84), Mutual Folding Induced by Binding (MFIB) (85), DisProt (55), molecular recognition feature (MoRF) (86), and FuzDB (87) databases. We filtered these databases for regions of IDRs that mapped to the AFDB (*Methods*) and were left with a total of ca. 61,000 residues for further analysis. Remarkably, AlphaFold2 assigned confident pLDDT scores (≥70) to 58.9% of all IDR residues in these databases, ranging from 35 to 87% when each database is analyzed separately (*SI Appendix*, Fig. S9). In comparison, only 14.3% of all residues predicted to be disordered by the SPOT-disorder predictor were assigned confident pLDDT scores (Fig. 1). Therefore, experimentally validated conditionally folded IDRs are enriched in confident and very confident AlphaFold2 pLDDT scores.

**Fig. 5.**
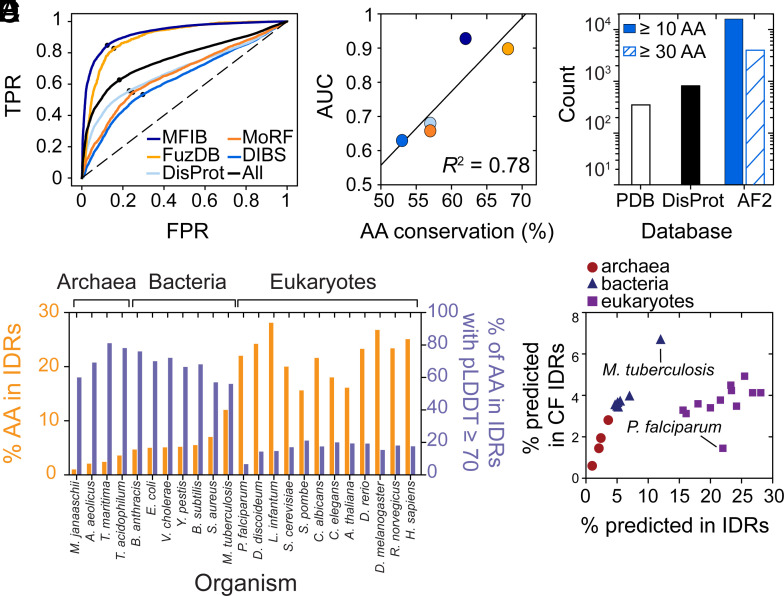
Systematic identification of conditionally folded IDRs in archaea, bacteria, and eukaryotes. (*A*) ROC curve for AlphaFold2 pLDDT-based classification of conditionally folded IDRs based on databases of known examples (MFIB, FuzDB, DisProt, MoRF, DIBS). The AlphaFold2-performance on the binary classification task (conditional folder/non-conditional folder) is displayed, with TPR (FPR) corresponding to true (false) positive rate. All five databases were merged (All, *black*) The AUC is 0.76 for the combined dataset. The black dot on each curve represents the threshold at which the TPR-to-FPR ratio is largest. (*B*) Correlation between the mean positional amino-acid conservation of IDR sequences from the databases listed in panel *A* and the AUC values from the ROC curves in panel *A*. The best-fit line is shown in black and has a Pearson’s *R*^2^ of 0.78. Note that gaps in the sequence alignments were ignored for the calculation of positional conservation. (*C*) IDRs with pLDDT scores greater than or equal to 70 for continuous regions of 10 or 30 or more amino acids are shown in blue and white with blue lines, respectively. For comparison, the number of conditionally folded IDRs in DisProt (black) and the PDB (white) are shown. (*D*) For each species listed, the percentage of disordered residues in the proteome (predicted by IUPred2A) is shown in orange on the left *y* axis. The percentage of predicted disordered residues with pLDDT scores ≥ 70 (i.e., conditionally folded IDRs) is shown in blue on the right *y* axis. (*E*) The percentage of predicted disordered residues in the proteome of each organism from panel *D* plotted against the predicted percentage of residues in predicted IDRs with pLDDT scores greater than or equal to 70, conditionally folded (CF) IDRs.

Next, we wondered whether the pLDDT scores from AlphaFold2 can be used to differentiate between IDRs that conditionally fold and those that remain disordered. To test this quantitatively, we assessed the classification potential of AlphaFold2 with a receiver operating characteristic (ROC) analysis. We extracted the conditionally folded IDRs from the above-mentioned databases as true positives (ca. 61,000 residues from 1,400 IDRs). To obtain a dataset of true negatives, i.e., IDRs that do not conditionally fold, we filtered the CheZOD database of proteins with assigned NMR chemical shifts (88) to exclude IDRs that have been reported to conditionally fold or show sequence homology to the PDB (*Methods*). We were left with ca. 8,200 residues from ca. 500 NMR-validated IDRs that are not known to conditionally fold (*SI Appendix*, Figs. S10 and S11). We then tested the ability of AlphaFold2 to classify conditionally folded IDRs based on pLDDT scores alone, finding that the ROC analysis yields values of AUC (area under the curve) between 0.63 and 0.93 depending on the input set of true positives (Fig. 5*A* and *SI Appendix*, Table S5). When all of the ca. 1,400 IDRs that are known to conditionally fold are supplied as input, we find that AlphaFold2 successfully classifies the conditionally folded IDRs with an AUC of 0.76 (Fig. 5*A*). Further, we observed a correlation between the average positional amino-acid sequence conservation of IDRs in each database and the classification performance (AUC) (Fig. 5*B*). In other words, the somewhat lower pLDDT scores for conditionally folded IDRs in the DIBS, DisProt, and MoRF databases may reflect higher conformational plasticity enabled by the reduced constraint on positional conservation. Overall, this ROC analysis is consistent with our hypothesis that IDRs/IDPs with confident and very confident pLDDT scores are likely to be conditional folders (Fig. 5*A*).

### AlphaFold2-Enabled Identification of Conditionally Folding IDRs in Other Organisms.

Based on our finding that the pLDDT score of AlphaFold2 enables identification of conditionally folded IDRs, we sought to use AlphaFold2 to identify more IDRs that conditionally fold. In humans, there are ca. 800 IDRs that are known to conditionally fold (Fig. 5*C*) (55, 84, 85). Acknowledging the caveats related to SPOT-Disorder false positive rates and the challenges in classifying IDRs and different types of disorder, as well as the curation of IDR databases, AlphaFold2 has significantly expanded the structural coverage of conditionally folding IDRs. Quantification of the false positive rate on these predictions is challenging, predominantly due to the inherent biases and difficulties in assembling databases of (non-)conditionally folding IDRs, including our filtering of the CheZOD database of NMR-validated IDRs. Moreover, given that AlphaFold2 was not specifically trained to identify conditionally folded IDRs, and saw very few structural examples of conditionally folded IDRs while training on the PDB, it remains remarkable that AlphaFold2 can identify IDRs that acquire folds (Fig. 5*A*). This observation motivated us to quantify the extent of conditionally folded IDRs in other organisms.

Given that the percentage of intrinsically disordered residues in the proteome has increased from archaea to bacteria to eukaryotes (89), we wondered if the percentage of conditionally folded IDRs has also changed. To this end, we used IUPred2A (65) to predict the IDRs in other AFDBs that are publicly available, as the IUPred2A software program is ca. 100-fold faster than SPOT-Disorder and provides a balance between the calculation speed and accuracy of the prediction (54). We first compared the predicted number of disordered residues and conditionally folded IDRs in the human proteome, as obtained by using the IUPred2A and SPOT-Disorder predictions to filter the AFDB. We found that IUPred2A and SPOT-Disorder give comparable results: 32% vs. 25% of all residues are predicted to be disordered with 14.3% vs. 17.6% of predicted disordered residues having pLDDT scores greater than or equal to 70 for SPOT-Disorder and IUPred2A, respectively (*SI Appendix*, Tables S1 and S2). Thus, although IUPred2A underestimates the extent of disorder, the boost in calculation speed provides an attractive approach to perform more high-throughput calculations.

We extracted the IUPred2A-predicted IDRs from the 23 AFDBs shown in Fig. 5*D*, including four archaeal, seven bacterial, and 12 eukaryotic organisms. As expected, the percentage of disordered residues in the proteome increased from archaea to bacteria to eukaryotes, with minimum and maximum values of 1.0% and 28.1% obtained for *Methanocaldococcus janaaschii* and *Leishmania infantum*, respectively (Fig. 5*D*). Interestingly, the percentage of IDRs with high-confidence pLDDT scores showed an inverse relation with the overall disordered content. That is, organisms with fewer predicted IDRs have a higher proportion of IDRs with high-confidence pLDDT scores (Fig. 5*D*). This result suggests that conditionally folded IDRs are the dominant type of IDRs in the archaea and bacteria examined here, where the percentage of IDRs with high-confidence pLDDT scores ranges from 56% (*Mycobacterium tuberculosis*) to 81.1% (*Thermotoga maritima*). By contrast, in the eukaryotes analyzed here, conditionally folded IDRs appear to be the minority, with minimum and maximum values of 6.6% (*Plasmodium falciparum*) and 21.1% (*Schizosaccharomyces pombe*) (Fig. 5*D*). The inverse relation between the percentage of disordered residues in the proteome and the percentage of IDRs that conditionally fold suggests that an upper bound of ca. 5% of residues in the proteome localize to conditionally folded IDRs (Fig. 5*E*). Although these results on the percentage of IDRs in various proteomes with high pLDDT scores are empirical and presently without theoretical basis, they suggest that organisms with higher percentages of IDRs, and thus a lower fraction of IDRs that conditionally fold, do not functionally utilize IDRs predominantly in the context of conditional folding. Rather, the majority of IDRs in eukaryotic organisms likely function in the absence of conditional folding.

### Leveraging AlphaFold2 to Understand Disease-Causing Mutations in Conditionally Folding IDRs.

**Fig. 6.**
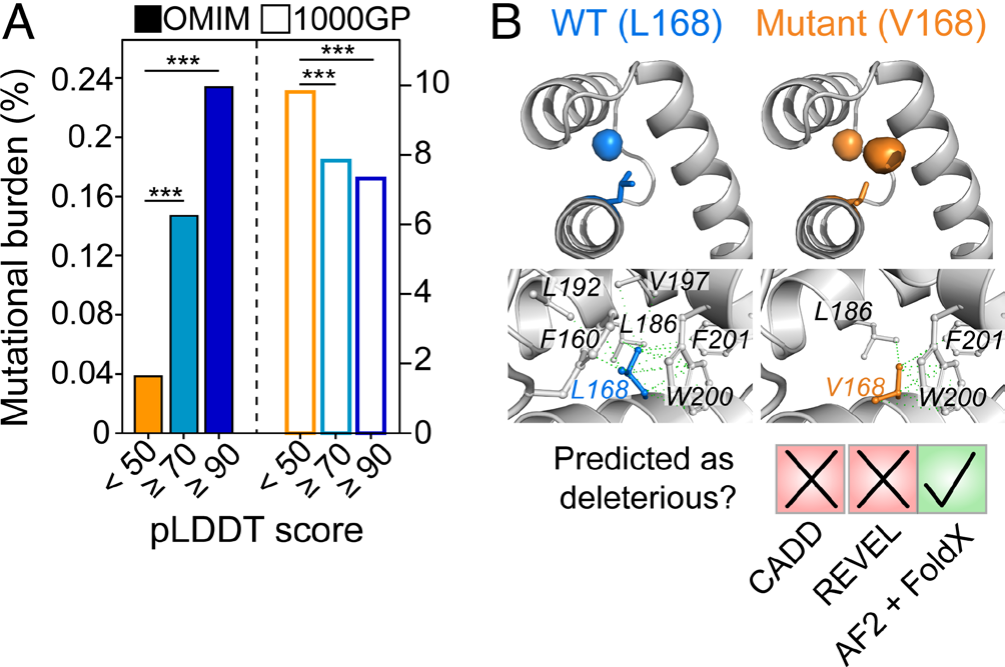
Using AlphaFold2 to understand the basis of disease-causing mutations in conditionally folded IDRs. (*A*) The per-residue mutational burden (the number of mutations divided by the total number of residues) for IDRs is shown as a function of AlphaFold2 pLDDT scores (<50, ≥70, ≥90). Disease-associated mutations from OMIM are shown in solid bars on the left, and presumably non-pathogenic mutations that are present in the general human population (1000GP) are shown in empty bars on the right. *** indicates a *P* value < 0.0001 from a Fisher Exact Test. (*B*) The L168V mutation in *ALX3* causes frontonasal dysplasia, but the mutation is predicted to be likely benign by CADD and REVEL. The high-confidence AlphaFold2 model shows that L168V creates a large cavity (orange spheres). In combination with FoldX, the AlphaFold2 model yields the prediction that L168V is severely destabilizing, with a ΔΔG value of 7.3 ± 0.1 kcal mol^−1^ relative to wild-type ALX3 (ΔΔG = ΔG_WT_–ΔG_mutant_). Hydrophobic interactions between the L168 side chain (blue) and other atoms in the ALX3 homeodomain that are within a 4.5-Å distance threshold are shown (green lines). A total of 26 interactions were identified. The residues involved in these interactions are indicated. In silico mutagenesis of L168 to Val was performed by FoldX. Hydrophobic interactions involving V168 and other atoms in the ALX3 homeodomain are indicated. Only 14 interactions are identified. Arpeggio was used for this analysis (92).

Next, we explored whether we could use AlphaFold2 predictions of conditional folding to obtain insight into disease-causing mutations in IDRs. We computed the per-residue mutational burden in IDRs for disease- *versus* non-disease-associated mutations as a function of the pLDDT score (Fig. 6*A*). To this end, we mapped the non-disease-associated sequence variations from the 1000 Genome Project (1000GP) (90) to human IDRs (*n* = 332,844 mutations) and calculated the per-residue substitution rates as a function of the range of pLDDT scores (Fig. 6*A*). The sequence variants within the 1000GP dataset should predominantly reflect presumably non-pathogenic mutations that have risen to appreciable frequency and are therefore polymorphic within the human population. We hypothesized that IDRs with low pLDDT scores, i.e., IDRs predicted not to conditionally fold, would be more tolerant to non-pathogenic mutations and therefore have a higher per-residue polymorphism rate than IDRs with high pLDDT scores. Indeed, we observed that IDRs with low-confidence AlphaFold2 scores (<50, *n* = 237,880 mutations) have a significantly higher per-residue polymorphism rate than IDRs with high (≥70, *n* = 31,415) or very high (≥90, *n* = 8,895) pLDDT scores (Fisher Exact Test, *P* < 0.00001 for both comparisons) (Fig. 6*A*). Thus, the absence of structural constraints for regions with low pLDDT scores is also reflected in their tolerance to substitutions and more rapid evolution than IDRs with high pLDDT scores (i.e., conditionally folded IDRs) (91).

Next, we mapped all known disease-causing mutations from the Online Mendelian Inheritance in Man (OMIM) database (93) to human IDRs (*n* = 1,963 mutations). We then calculated the per-residue mutational burden in IDRs that have very high (≥90, *n* = 286 mutations), high (≥70, *n* = 599), or low (<50, *n* = 960) pLDDT scores (Fig. 6*A*). In IDRs with high and very high pLDDT scores, we found a strong enrichment in disease-causing mutations relative to IDRs with low confidence scores (Fig. 6*A*). The per-residue mutational burden in IDRs with high and very high pLDDT scores was respectively increased by four- or sixfold relative to IDRs with low pLDDT scores (Fisher Exact Test: *P* < 0.00001 for both comparisons). This suggests that, for these disease-associated mutations in OMIM, the IDRs with high confidence AlphaFold2 scores are less tolerant of sequence changes, and that such substitutions are more likely to manifest as disease when compared to IDRs with lower pLDDT scores. Presumably, the constraint of acquiring a conditional fold limits the sequence space of these IDRs and increases the likelihood of a deleterious mutation (91). As a comparison, we also mapped OMIM mutations to PFAM domains that were filtered to exclude any SPOT-Disorder-predicted IDRs (*SI Appendix*, Fig. S12). As expected, many more OMIM mutations map to PFAM domains (*n* = 23,654 vs. 1,963 mutations in SPOT-Disorder-predicted IDRs), which reflects both the decreased tolerance of folded regions to amino-acid substitutions and the biases in studying disease-mutations in folded regions. For PFAM domains with very confident pLDDT scores, we find that the OMIM mutational burden is ca. twofold-to-fourfold higher than IDRs with pLDDT scores ≥ 90 and ≥ 70, respectively (*SI Appendix*, Fig. S12). Thus, conditionally folded IDRs show intolerance to amino-acid substitutions to an extent that falls between PFAM domains and non-conditionally folding IDRs.

Moving beyond the statistical association of disease mutations with predicted conditional folding, we next sought to leverage the high-confidence AlphaFold2 structural predictions to gain mechanistic insight into the effects of disease mutations on protein function. An illustrative example is the L168V mutation in the gene *ALX3*, which causes the rare disease frontonasal dysplasia (94). *ALX3* encodes for a transcription factor named homeobox protein aristaless-like 3 (ALX3, UniProt: O95076) that plays a key role in development (95). The L168V mutation maps to a DNA-binding homeodomain of ALX3 (residues 153 to 212), for which there is no experimental structure and SPOT-Disorder predicts to be intrinsically disordered. Indeed, the bulk sequence properties of the ALX3 homeodomain, such as mean hydrophobicity (0.37) and mean net charge (0.13), are indicative of an IDR (*SI Appendix*, Fig. S13) (80). Furthermore, other homeodomains are known to be marginally stable in the absence of DNA, e.g., the homeodomains from *Drosophila melanogaster* Engrailed and NK-2 have a free energy of denaturation of only 2.2 kcal mol^−1^ (96) and a melting temperature near 25 °C (97), respectively. The binding of DNA to the NK-2 homeodomain induces additional folding and significantly increases its stability (98). Thus, the amino-acid sequence of the ALX3 homeodomain may encode a conditionally folded IDR that acquires structure or enhanced stability upon binding to DNA.

Even if the ALX3 homeodomain conditionally folds, it remains difficult to rationalize how the chemically subtle L168V mutation might affect function. Indeed, two predictors of variant pathogenicity, the ensemble-based CADD (Combined Annotation Dependent Depletion) (99) and the meta-predictor REVEL (Rare Exome Variant Ensemble Learner) (100), classify the L168V mutation as “likely benign” (Fig. 6*B*). All residues of the ALX3 homeodomain (153 to 212) have pLDDT scores above 70, and residues 159 to 208 all have pLDDT scores above 95, indicating a very confident AlphaFold2 prediction with likely accurately positioned side-chain rotamers (3). Close inspection of the AlphaFold2 structural model reveals that the L168 side chain makes 26 different hydrophobic interactions with nearby aliphatic and aromatic residues from all three helices of the homeodomain (*SI Appendix*, Fig. S13), thus coordinating key interhelix contacts in the three-dimensional structure. Upon in silico mutation of L168 to Val, a total of 12 of these hydrophobic interactions are lost and a new hydrophobic cavity is created (Fig. 6*B*).

Since high-confidence AlphaFold2 models perform as well as if not better than experimental X-ray structures for structure-based protein stability calculations (8), we used the high-confidence AlphaFold2 model of the conditionally folded state of ALX3 to calculate protein stability using FoldX (101) (Fig. 6*B*). Indeed, the predicted stability of L168V is significantly lower than the wild-type protein, with a FoldX-determined ΔΔG of 7.3 ± 0.3 kcal mol^−1^, nearly 10-fold larger than the error associated with FoldX predictions (Fig. 6*B*) (101, 102) and much larger than typical ΔΔG values [1 ± 2 kcal mol^−1^ (103)]. Thus, the ΔΔG value of 7.3 ± 0.3 kcal mol^−1^ observed for L168V in ALX3 is expected to be highly destabilizing. Given that a positive ΔΔG value indicates an increased population of an unfolded or partially folded state, a plausible outcome of the L168V mutation is that the ALX3 homeodomain remains disordered or no longer properly folds upon binding to DNA, thereby preventing or altering its transcriptional activity. Although the predicted destabilization of ALX3 with the L168V mutation awaits experimental validation, a Leu-to-Val mutation in the homeodomain of a related protein (SHOX) at the same position as L168 in ALX3 was shown to abolish dimerization and DNA binding (104). Our analysis of ALX3 provides a mechanistic hypothesis about how a disease mutation may disrupt the function of ALX3, namely by destabilizing the conditionally folded DNA-bound state.

## Discussion

The application and development of machine learning methods in structural biology has revolutionized the field of protein structure prediction (3–5). AlphaFold2 can predict the structures of most globular proteins to near atomic-level accuracy (3). However, approximately 30% of the human proteome is intrinsically disordered, with over 60% of all human proteins containing at least one IDR longer than 30 residues in length (17, 19). Thus, it is essential to critically analyze the AlphaFold2-predicted structures of IDRs, as such regions cannot be accurately described by a single, static structure (21).

Here, we have shown that there are thousands of predicted IDRs in the human proteome that are ascribed confident or very confident pLDDT scores in the AFDB (Fig. 1*A*) and thus have confidently predicted three-dimensional structures. These high-confidence AlphaFold2 structures of IDRs often can capture a folded conformation that forms in the presence of a specific binding partner or upon PTM (Figs. 2 and 3 and *SI Appendix*, Fig. S3), even though the AlphaFold2 structures were predicted in the absence of such binding partners or PTMs. AlphaFold2 assigns confident pLDDT scores to these IDRs likely due to constraints imposed by their amino-acid sequences, and not PDB templating, as we find that 96% of the IDR sequences with high confidence AlphaFold2 structures do not have appreciable sequence similarity to the PDB (Fig. 4*C*). Thus, AlphaFold2 has likely identified conditionally folded IDRs through the co-evolution of specific residues, which can indicate spatial proximity within a three-dimensional structure (105–109). Indeed, co-evolution can recover the folded structures of some conditionally folding IDRs (83, 110). In conditionally folded IDRs, the importance of input MSAs is additionally demonstrated by the observed discrepancy between the performance of AlphaFold2 versus the Google Colaboratory versions (*SI Appendix*, Fig. S1).

Expanding our analysis of conditionally folded IDRs, we further found that AlphaFold2 can classify conditionally folded IDRs based solely on the per-residue pLDDT score (Fig. 5*A*). The performance of AlphaFold2 as a classifier of conditional folding depended on the input IDRs, with those in the MFIB (AUC = 0.93) and the DIBS (0.63) as the extrema. One possible explanation for the difference in classification performance is that the conditionally folded IDRs that AlphaFold2 struggles to identify might be more conformationally dynamic or sample more than one structure in the conditionally folded state. If so, there may be less of an evolutionary constraint for stable structure, which may lower the final pLDDT scores assigned to such regions. In such situations, the observed pLDDT scores in a conditionally folding IDR may be below the confidence threshold used here and not detected by our approach. AlphaFold2 predictions of small regions that are excised from a larger, full-length protein can yield different structures and higher pLDDT scores (111), which raises the possibility that more conditionally folded IDRs may exist that are presently not detected by pLDDT scores obtained from the full-length protein. On the other hand, one could speculate that the predicted IDRs with high pLDDT scores could be folded domains that are incorrectly annotated as IDRs by SPOT-Disorder; however, it is unlikely that mis-annotated folded domains are responsible for the observed pLDDT scores, as only 4% of the predicted IDRs with high pLDDT scores have any appreciable sequence similarity to the PDB. Folded domains, which exhibit more positional sequence conservation than IDRs, would be expected to yield significantly more hits to related domains in the PDB. Furthermore, a recent comparison of experimental NMR data and about 25 different disorder predictors found that SPOT-Disorder is the most accurate predictor of disorder and the best discriminator between order and disorder, while also slightly underestimating the extent of disorder (112). Thus, false-positive predicted IDRs likely are not a significant factor in our analyses of conditionally folding IDRs. A more significant source of error likely arises from biases present in the curation of databases of IDRs and (non-)conditionally folding IDRs.

A valuable metric that emerges from our analyses is an estimate on the fraction of IDR residues that conditionally fold (Fig. 5*C*). There are presently ca. 800 known human IDRs that conditionally fold, based on manual curation of the DisProt database. Thus, the ca. 15,000 human IDRs longer than 10 residues with high-confidence AlphaFold2 structures provide a useful resource to interrogate the sequence and structural properties of IDRs that acquire folds during their function. At present, we do not know the rate with which AlphaFold2 incorrectly predicts conditionally folded IDRs; however, noting that AlphaFold2 correctly identified ca. 60% of IDRs that are known to conditionally fold over five different databases, we can estimate an upper bound of conditionally folded human IDRs at 25% (0.15/0.60). A percentage of conditionally folded human IDRs between 15 and 25% correlates with previous observations that a minority of human IDRs display significant degrees of positional sequence conservation (76), with many of the positionally conserved IDRs identified as those that fold upon binding (76, 81). Positional conservation is atypical of IDRs that generally evolve rapidly and show low positional conservation, even though there is significant conservation of bulk molecular features (78, 113). Therefore, there may be purifying selection upon IDR sequences that function by conditional folding, such that the sequence only slowly evolves in order to maintain the overall fold of the bound/modified form of the IDR.

Collectively, these results lead to the hypothesis that if only 15 to 25% of human IDRs are conditionally folded, then the majority of IDRs in the human proteome, and those of other eukaryotes, would function in the absence of stable structure. These would include IDRs involved in discrete dynamic or “fuzzy” complexes (26, 36, 37) and those with low complexity sequences that participate in dynamic, exchanging condensed phases of biomolecular condensates (38). On the other hand, our analysis of the percentage of conditionally folded IDRs in various organisms reveals that archaea and bacteria, which have lower disordered content throughout the proteome (89), have a relatively higher percentage of IDRs that conditionally fold (Fig. 5 *F* and *G*). In prokaryotes, therefore, the majority of IDRs seem to conditionally fold. We also emphasize that, if a protein is disordered in isolation but always folded in a complex, otherwise known as conditionally disordered (114, 115), then we would consider this protein to be conditionally folded. Prokaryotes may contain more conditionally folded IDRs that exist as obligate multimers, for example, although this remains to be explored.

Our finding that AlphaFold2 has learned to identify conditionally folded IDRs will enable insights into the sequences, evolution, and structural bioinformatics of these regions. Along with other recent analyses of IDRs (21, 52, 116, 117), our work demonstrates that AlphaFold2 has learned interesting properties about certain IDRs. While the structural predictions generated by AlphaFold2 will certainly accelerate the pace of biomedical discovery, there remains a huge need for experimental (22) and bioinformatic (78, 113, 118) approaches to address the majority of IDRs that likely function in the absence of folded structure. With increased experimental data on IDRs/IDPs, including integrative structural modeling (43–46), machine-learning methods promise to provide insights into disordered protein conformational states and functional mechanisms (119). The complementary nature of AlphaFold2 structural predictions and atomic-level insight from NMR spectroscopy will be pivotal for future developments related to IDR conformational ensembles.

## Methods

### Sequence-Based Prediction of IDRs in the Human Proteome.

We obtained all protein sequences in the human proteome from the UniProt database (reference proteome number UP000005640, downloaded in November 2021). This reference human proteome contains 20,959 unique UniProt IDs that have a total of 11,472,924 residues. To identify IDRs in the human proteome, we used SPOT-Disorder version 1 (53), which was recently identified as one of the most accurate predictors of disorder (54) and gave the closest agreement with experimentally determined disordered regions based on NMR data (88, 112). SPOT-Disorder version 1 values of 0.5 or higher were considered to be disordered. Regions of the proteome that were not predicted to be disordered were assumed to be ordered. For analysis of the per-residue pLDDT scores, the SPOT-Disorder predictions were used without filtering for consecutive residue length (Fig. 1*A*); in the bioinformatic analyses of Fig. 4, to exclude very short segments, we filtered the SPOT-Disorder predictions to include only the regions with predicted consecutive disorder greater than 10 residues. For the analysis of sequence-based predictors of disorder in Fig. 2*A*, we used the software packages metapredict, SPOT-Disorder, DISOPRED3, and IUPred2A (53, 64–66). SPOT-Disorder was run as noted above. The webserver versions of the other three software programs were used with default parameters.

The remaining methods are described online in *SI Appendix*.

## Supplementary Material

Appendix 01 (PDF)

## Data Availability

The code and data used in this paper are available on GitHub (https://github.com/IPritisanac/AF2.IDR) (120). The AlphaFold EMBL-EBI website was used to download AFDB files (https://alphafold.ebi.ac.uk/) (121). The specific UniProt (https://www.uniprot.org/) (122), PDB (https://www.rcsb.org/) (123) and BMRB IDs (https://bmrb.io/) (124) that were used in this work are listed in the text.
