# Supplementary material for: Systematic identification of conditionally folded intrinsically disordered regions by AlphaFold2
Source: Proc Natl Acad Sci U S A. 2023 Oct 25;120(44):e2304302120. doi: 10.1073/pnas.2304302120 (PMC10622901; doi:10.1073/pnas.2304302120)
Supplement: Supplementary file 1 — Appendix 01 (PDF) [file pnas.2304302120.sapp.pdf]

Supplementary Information for:

Systematic identification of conditionally folded intrinsically disordered regions by AlphaFold2

T. Reid Alderson[1,2§], Iva Pritišanac[3,4,5,§], Đesika Kolarić[5], Alan M. Moses[3], Julie D. Forman-Kay[1,4*]

1. Department of Biochemistry, The University of Toronto, ON, Canada M5S 1A8
2. Department of Molecular Genetics, The University of Toronto, ON, Canada M5S 1A8
3. Department of Cell and Systems Biology, University of Toronto, Toronto, Canada
4. Program in Molecular Medicine, The Hospital for Sick Children, Toronto, ON, Canada M5G 1X8
5. Gottfried Schatz Research Center for Cell Signaling, Metabolism and Aging, Molecular Biology and Biochemistry, Medical University of Graz, 8010 Graz, Austria

[§] Equal contribution

* Correspondence to forman@sickkids.ca

Supplementary Methods

Extraction of per-residue pLDDT scores from the AFDB

Per-residue pLDDT scores were extracted from each PDB file in the database using an in-house Python script that is available on GitHub (https://github.com/IPritisanac/AF2.IDR), as are the scripts mentioned below. The per-residue SPOT-Disorder predictions of disorder were then mapped onto the AFDB in order to split the AlphaFold2 data into predicted regions of disorder and order. In this way, our analysis should not be biased by the assignment of secondary structure in the AlphaFold2-generated structures; instead, we relied on SPOT-Disorder to identify the IDRs and ordered regions. The SPOT-Disorder-predicted regions of disorder were further split into the AlphaFold2-designated levels of confidence: very low (≤ 50), low (≤ 70), confident (≥ 70 *x* < 90), or very confident (≥ 90) pLDDT scores.

For the organisms listed in **Figure 6**, AFDBs were downloaded from the AlphaFold website in January 2022. For organisms that did not have pre-compiled AFDBs at that point, but did have AlphaFold2 structures available, an in-house Python script was used to automatically download all structures that matched query UniProt IDs from a UniProt proteome file.

PDB files within the AFDB

The repository of human protein structures was downloaded in November 2021 from the AlphaFold Protein Structure Database using the reference proteome number UP000005640. This corresponded to version 1 of the human AFDB, as indicated with the “v1” string in the name of the downloaded file. The database contained 23,391 predicted structures that map to 20,504 unique UniProt IDs, which corresponds to 97.8% of the proteome (containing 20,959 unique entries). The total number of residues in the AFDB is 10,825,508, which is 94.4% of all residues in the proteome (11,472,924 residues) and *ca.* 2.6% more than what was reported in the original work (1), likely reflecting the *ca.* 1% increase in the number of UniProt IDs (20,504 in AFDB *vs.* 20,296 sequences reported in the original work).

In the AFDB, the greater number of structures (23,391) relative to the number of unique UniProt IDs (20,504) is due to the 2,700-residue limit for AlphaFold2 structures: proteins longer than this threshold are segmented into multiple structures that contain overlapping 1,400-residue fragments (*e.g.*, residues 1-

1400, 201-1600, etc.). The human proteome contains 210 proteins longer than 2,700 residues; searching the AFDB for the UniProt IDs that map to these proteins reveals 3,095 AlphaFold2 structures, accounting for the difference in the number of PDB files and unique UniProt IDs.

Biological Magnetic Resonance Bank

Assigned NMR chemical shifts for the IDRs/IDPs α-synuclein, 4E-BP2, and ACTR were downloaded from the BMRB (2) using the following entry identification numbers: 6968 (α-synuclein) (3), 5744 (α-synuclein bound to SDS micelles) (4), 19114 (4E-BP2) (5), 19905 (phosphorylated 4E-BP2) (6), 15397 (ACTR) (7), and 5228 (ACTR bound to CBP) (8). All of the entries contained assignments for ^{1}HN, ^{1}Hα, ^{15}N, ^{13}CO, ^{13}Cα, and ^{13}Cβ chemical shifts, except for 5744 (no ^{1}Hα and ^{13}CO assignments) and 15397 (no ^{1}Hα assignments).

Calculation of NMR chemical shifts from an input PDB structure

To simulate the NMR chemical shifts of AlphaFold2-generated structure predictions, we used the SPARTA+ software package (9). Protons were first added to each PDB structure using DYNAMO version 7.2 available via the PDB Utility Web Servers from the Bax Laboratory (https://spin.niddk.nih.gov/bax/nmrserver/pdbutil/sa.html). The proton-containing PDB files were then uploaded to the SPARTA+ Web Server (https://spin.niddk.nih.gov/bax/nmrserver/sparta/) using default parameters. The backbone and $^{13}C^{\beta}$ chemical shifts were extracted from the output file with an in-house Python script.

Calculation of secondary structure propensity from NMR chemical shifts

We used the SSP software program (10) via the NMRbox (11) to calculate the secondary structure propensity (SSP) of the IDRs/IDPs shown in **Figure 2**. We included $^{13}C^{\alpha}$ and $^{13}C^{\beta}$ shifts as input, as recommended in the original SSP publication. Prior to analysis, we re-referenced all of the chemical shifts using standard protocols (10). This is important because secondary chemical shifts, and therefore SSP, are highly sensitive to the internal referencing of the measured chemical shifts, and any errors in referencing will impact the downstream SSP analysis. The SSP-derived re-referencing offset ppm in the ^{13}C dimension

for each dataset was -0.390 (BMRB: 6968), -0.483 (5744), +0.373 (191141), +0.106 (19905), -0.051 (15397), and -0.139 (5228).

Sequence similarity between IDRs and the PDB

We ran BLASTP (12) to determine to which extent IDR sequences with different pLDDT scores overlap with sequences in the PDB. To filter the sequences that are in the PDB, we removed duplicates of identical sequences and homologous sequences using PISCES (13) with the following parameters: maximum pairwise percent sequence identity (75%), resolution (0-3.5A), minimum chain length (40), and the maximum chain length (10,000). The sequences included all X-ray structures that met the aforementioned criteria but excluded all NMR and cryo-EM entries as well as sequences with breaks or those that map to regions with no electron density. We separately downloaded all X-ray structures that contain 40 or fewer residues from the PDB, and these were combined with the PISCES-filtered sequences to create the BLAST database. BLASTP was run with E-value cut-off values of 1e-3 and 1e-6, with restrictions on sequence identity (>30%) and coverage criteria (>60%). The percentage of IDR sequences with homologs in this BLAST database was 4%. We also tested a BLASTP database that included all non-redundant sequences, including NMR structures and those that included breaks or regions with no density. The percentage of IDR sequences with confident pLDDT scores that had homologs in the non-redundant PDB was 9%, although this is likely an overestimate since regions without density may contribute to this value and NMR structures were not used for the training of AlphaFold2.

Evaluation of positional sequence conservation in IDRs

Positional sequence conservation was computed for alignments of IDRs that were distributed in three sets with different cut-offs of pLDDT scores (see "Mapping pLDDT scores to IDRs"). Only IDR sequences with 10 or more residues with consecutive pLDDT score below or above the desired threshold were considered. To compute positional conservation across MSA columns, we used a modified metric of Shannon’s entropy, the so-called property entropy as introduced by Capra and Singh (14). Gaps were ignored in the computation of positional conservation.

Bioinformatic analysis of the predicted IDRs in the AFDB

There are 10,825,508 residues in the AFDB, of which 3,539,799 are predicted by SPOT-Disorder to be disordered (**Supplementary Table 1**), 7,127,685 to be ordered, and 158,024 are not mapped. The latter is consistent with the *ca.* 98.5% coverage of the human proteome in the AFDB (1). From these numbers, we can also calculate the percentage of residues in SPOT-Disorder-predicted IDRs, which amounts to 32.7% of the AFDB, in agreement with literature values (15).

Next, the SPOT-Disorder-predicted IDRs were further split into those with low pLDDT scores (< 70) and those with high pLDDT scores (≥ 70). A total of 506,101 residues are in $\text{IDR}_{\text{high pLDDT}}$ (pLDDT ≥ 70) as compared to 3,033,698 in $\text{IDR}_{\text{low pLDDT}}$ (pLDDT < 70). Using an in-house Python script, we calculated the amino-acid frequencies in each of the following categories: ordered regions, IDRs with low pLDDT scores ($\text{IDR}_{\text{low pLDDT}}$), and IDRs with high pLDDT scores ($\text{IDR}_{\text{high pLDDT}}$). The amino-acid frequencies were normalized to the total number of amino acids in each category to yield the percentage of the total:

$$AA_{\text{freq,i,j}} = AA_{i,j} \Big/ \sum_{i=1}^{20} AA_{i,j} \qquad (1)$$

Where $AA_{\text{i,j}}$ stands for the number of amino acids of residue type i, with the indices i and j respectively indicating the amino acid type (A, C, D, … , V, W, Y) and the category of residues analyzed (ordered, $\text{IDR}_{\text{low pLDDT}}$, $\text{IDR}_{\text{high pLDDT}}$). The summation in the denominator of eq 1 refers to the total number of amino acids in each category.

Mean net charge and mean hydrophobicity of $\text{IDR}_{\text{low pLDDT}}$ and $\text{IDR}_{\text{high pLDDT}}$ sequences were calculated according to Uversky *et al.* (16) using an in-house Python script. Briefly, the net charge of a given IDR/IDP sequence was computed at pH 7. The pK_a of histidine residues was set to 6.5 to match an experimental determination of the His pK_a in an IDP recorded in the presence of physiological salt concentration (17). The absolute value of the net charge of the IDR/IDP was then divided by the total number of residues to obtain the mean net charge. The mean hydrophobicity of an IDR was computed using a normalized version of the Kyte-Doolittle hydropathy scale, such that the values ranged between 0 and 1. The hydropathy value of each residue in the IDR/IDP was then averaged over a sliding window of five residues. The mean hydropathy was finally obtained by computing the sum of all hydropathy values (via the sliding window) and then dividing by the total number of residues.

Databases of IDRs/IDPs that fold upon binding

To determine if AlphaFold2 can systematically identify IDRs/IDPs that conditionally fold, we first extracted the amino-acid sequences of IDRs/IDPs that are known to conditionally fold (i.e., true positives). To this end, we filtered five databases that contain manually curated lists of IDRs/IDPs that conditionally fold: DisProt (Quaglia et al 2022), FuzDB (Hatos et al 2021), MoRF (18), and the previously mentioned DIBS (Schad et al 2018) and MFIB (Fichó et al 2017). Then, in order to extract the per-residue pLDDT scores of these IDR/IDP sequences, an in-house Python script was written to download and filter AlphaFold2 structural models from the AFDB. From the DIBS and MFIB databases 551 and 253 regions accounted for 9,351 and 20,482 residues, respectively. Within the DisProt database, we only considered IDRs that have the annotation "structural transition: disorder-to-order", which generated a total of 473 regions and 27,013 residues with corresponding pLDDT scores for further analysis. Finally, from 95 regions in the FuzDB that are listed as "disorder-to-order regions" (DOR), 757 pLDDT scores of corresponding residues were extracted for further analysis. The output files contained the UniProt IDs, pLDDT scores, amino-acid types, and residue numbers of the IDRs/IDPs that were taken from each database.

Next, we compiled a curated dataset of IDRs/IDPs that have not been reported to fold upon binding (i.e., true negatives). Assembling a true negative set, however, is a challenging task due to experimental biases and the low-throughput in experimental characterization of IDR conformational landscapes. For example, some IDRs that conditionally fold may have yet to be studied under the conditions that promote folding. Thus, although we refer to the dataset as "true negatives", these IDRs have been experimentally validated and filtered to exclude any known conditional folders, yet this remains an imperfect dataset. Previous software programs that were specifically designed to detect disordered binding regions were trained on a list of flexible linkers between structured domains (18–20). However, when we examined the AlphaFold2 pLDDT scores of these flexible linkers (n = 4,765 residues from 386 regions), we found that the majority of these regions are short (e.g., fewer than 10 residues) and highly conserved with high or very high pLDDT scores (**Supplementary Figure 10**) For these reasons, we constructed a new dataset of true negatives using NMR data from IDRs that have not been reported to conditionally fold. We used the CheZOD database (Nielsen & Mulder 2016) to identify IDRs/IDPs that do not conditionally fold. Importantly,

CheZOD contains a manually curated and filtered list of proteins with assigned NMR chemical shifts that are used to experimentally quantify (dis)order at the residue level (21).

The expanded version of the CheZOD database contains experimental NMR data from 1325 protein sequences (22). Since CheZOD was trained on both ordered and disordered sequences, we first removed regions with secondary or tertiary structure (Z-score > 3.0). We filtered this set of sequences to retain only those residues that have Z-scores below 3.0, which is indicative of disorder, as recommended by the developers (22). To extract only the unstructured regions, we then matched the remaining Z-scores (< 3.0) and residues with their UniProt IDs for regions longer than five residues. Finally, given that CheZOD contains protein sequences with associated NMR data, and thus is agnostic to the conditional folding of IDRs/IDPs, we excluded from CheZOD any sequence that overlaps with any of the five databases of IDRs/IDPs that conditionally fold, as well as any sequence with homology to sequences in the PDB (including cryo-EM and NMR structures). For this latter analysis, BLASTP was run using non-redundant sequences obtained from the PISCES (13) webserver (pdbnr.aa) and an E-value threshold of 1e-6. A total of 228 regions were identified, and the associated PDB matches were manually examined to find regions that contain coordinates in the PDB file and overlap with the query sequence boundaries. After projecting those regions to their associated AlphaFold2 structural models, a total of 498 IDRs and 8,202 pLDDT scores were extracted for further analysis. Despite our stringent filtering, some sequences within this dataset may conditionally fold (false negatives), but this cannot be avoided. We found that AlphaFold2 assigns low-confidence pLDDT scores (< 70) for 86% of the filtered CheZOD database (7,009 out of 8,2002 residues), which resembles the proportion of IDRs in human proteome that are given low-confidence pLDDT scores (~85%, **Figure 1B**). Moreover, the IDRs within the filtered CheZOD database have a similar median length but lower average alignment depth than IDRs in the flexible linkers dataset (**Supplementary Figure 10**). In other words, the IDRs within the flexible linkers dataset are more positionally conserved than those in the filtered CheZOD database.

Finally, we used these true negative (filtered CheZOD) and true positive databases (MFIB, FuzDB, DIBS, DisProt, MoRF) to assess the performance of AlphaFold2 on identifying IDRs/IDPs that conditionally fold. We used the pLDDT scores of the extracted regions that conditionally fold from each database as a true positive dataset, whereas the pLDDT scores of regions extracted from CheZOD database that do not

conditionally fold were used as a true negative dataset. The expected pLDDT scores were all set to 1 for the true positive dataset and to 0 for the true negative dataset. We then plotted ROC curves by comparing the observed pLDDT scores (normalized between 0 and 1) from the AlphaFold2 structural predictions against the expected pLDDT scores (0 or 1). The associated AUC, precision, and recall values for each true positive database are listed in **Supplementary Table 5**.

We ran the Anchor2 software with the default parameters on all sequences in the aforementioned databases (20). Anchor2 was originally developed to identify regions in IDRs that bind to other proteins, including those that undergo a disorder-to-order transition. Although not specifically designed to detect conditional folding, we used Anchor2 to compare to AlphaFold2 for the purpose of identifying IDRs that fold in the presence of a binding partner or upon PTM. We then performed an ROC analysis as above in which the values for IDRs in the same true positive and negative datasets were set to 1 and 0, respectively. Except for the DIBS database (AUC 0.59, precision 0.57, recall 0.61), which was used in the training of Anchor2, the classification of conditionally folded IDRs remains a challenging task for Anchor2. Overall, our classification comparison shows that, even though AlphaFold2 was never specifically trained to detect conditional folding or possible binding sites within IDRs, it performs well on this task, regardless of the differences in types of IDRs from different databases (**Supplementary Figure 11**).

Simulation of biophysical parameters from an input PDB structure

As outlined in **Supplementary Figure 4, Supplementary Figure 5,** and the **Supplementary Appendix**, biophysical experiments can be performed and compared to predictions derived from the AFDB structural model. Such comparisons would be able to rapidly report on the accuracy of the model relative to the conformations sampled in solution.

Circular dichroism (CD): CD spectra of proteins are sensitive to global secondary structure content and do not require much sample. The webserver PDB2CD (23) was used with default parameters to simulate the CD spectrum of the AFDB structure of human α-synuclein and to compare to experimental data (24).

Translational diffusion (PFG-NMR): The software package HYDROPRO was used to calculate hydrodynamic properties of α-synuclein based on its structure in the AFDB (25) (**Supplementary Figure 4B**). Unless otherwise specified, default parameters were used (*e.g.,* a non-overlapping shell model was used with the shell model set to the atomic level and a 2.84-Å radius of atomic elements). The temperature was set to 15 °C to match the experimental conditions (26), and the solvent viscosity was adjusted accordingly to 1.1366 cP based on the value reported by NIST (https://webbook.nist.gov/chemistry/fluid/). The AlphaFold2 structure from the AFDB was then loaded and the calculation was run. The predicted translational diffusion coefficient from HYDROPRO is 5.141 x 10^{-11} m^2 s^{-1}, which is approximately 10% smaller than the experimentally measured value of 5.71 ± 0.02 x 10^{-11} m^2 s^{-1} (26), suggesting that the AlphaFold2 structure is more extended than the conformation of α-synuclein observed experimentally. To generate a simulated plot of signal decay (I_j / I_0) caused by translational diffusion during a BPP-LED pulse sequence, equation 2 below was used:

$$I_j = I_0 \, e^{-\gamma^2 G_j^2 \delta^2 \left(\Delta - \frac{\delta}{3} - \frac{\tau}{2}\right) D} \qquad (2)$$

Where the measured signal intensity, I_j, depends on the exponential term above that contains the square strength of the applied gradient (G_j^2), which is varied during the experiment, and a linear contribution from the translational diffusion coefficient (D). The other parameters are either held fixed in the experiment (Δ, the delay time for translational diffusion; δ, the total duration of the encoding gradients; τ, the gradient recovery duration) or are physical parameters (γ, the gyromagnetic ratio of ^{1}H). Values of 267,522,187.44 rad s^{-1} T^{-1}, 200 ms, 3 ms, 200 μs, and 0.668 T m^{-1} were used for γ, Δ, δ, τ, and G_{max}, respectively, to match experimental conditions (26). The values of G_j were varied over a range of G_j / G_{max} from 0 to 1.

Small-angle X-ray scattering (SAXS): The software program Crysol (27) was used to simulate SAXS data of human α-synuclein based on the AFDB structure (**Supplementary Figure 4C**). Experimental SAXS data are available for comparison (28). The software package ATSAS (29) was used to analyze the SAXS data to obtain the radius of gyration (R_g) and the maximum distance (D_{max}). The fitted experimental values of R_g and D_{max} are 35.6 ± 0.2 Å and 109 Å, and those derived from the simulated data from the AFDB structure are 42.6 Å and 152 Å, respectively.

Other NMR parameters: The $^{13}C\alpha$ chemical shifts in **Supplementary Figure 4D** were simulated with SPARTA+ (9) after protons had been added to the AFDB structure of human α-synuclein and neighbor-corrected random coil chemical shifts were obtained from the SPARTA+ output file. The measured $^{13}C\alpha$ chemical shifts were extracted from (3). The $^{3}J_{HNH\alpha}$ coupling constants in **Supplementary Figure 4E** and **Supplementary Figure 5** were simulated from the AFDB structure of human α-synuclein in which protons had been added, as described above. The parameterized form of the Karplus equation used to relate the dihedral angle Φ to the $^{3}J_{HNH\alpha}$ coupling constant is shown below and based on (30):

$$^{3}J_{\mathrm{HNH\alpha}} = 7.97\cos\theta^{2} - 1.26\cos\theta + 0.63 \qquad (3)$$

Where θ is the dihedral angle Φ minus 60°, which is then converted to radians. Dihedral angles were computed from the AFDB structure of human α-synuclein using an in-house Python script that uses the BioPython package (31). The simulated values of $^{3}J_{HNH\alpha}$ were compared to those measured experimentally (32). Finally, $^{1}H\alpha$ solvent paramagnetic relaxation enhancements (sPREs) were obtained from (33). Simulated sPREs were performed using the sPRE-calc software program (34), and the simulated rates were then scaled to roughly match the lowest experimentally reported sPRE values.

AlphaFold Structures from the Proteomes of Other Organisms

UniProt Proteome files were downloaded from UniProt (https://www.uniprot.org/) for *Aquifex aeolicus* (UP000000798), *Arabadopsis thaliana* (UP000006548), *Bacillus anthracis* (UP000000594), *Bacillus subtilis* (UP000001570), *Candida albicans* (UP000000559), *Caenorhabditis elegans* (UP000001940), *Dictyostelium discoideum* (UP000002195), *Drosophila melanogaster* (UP000000803), *Danio rerio* (UP000000437), *Escherichia coli* (UP000000625), *Homo sapiens* (UP000005640), *Leishmania infantum* (UP000008153), *Methanocaldococcus jannaschii* (UP000000805), *Mycobacterium tuberculosis* (UP000001584), *Neurospora crassa* (UP000001805), *Plasmodium falciparum* (UP000001450), *Rattus norvegicus* (UP000002494), *Saccharomyces cerevisiae* (UP000002311), *Schizosaccharomyces pombe* (UP000002485), *Staphylococcus aureus* (UP000008816), *Synechocystis sp.* (UP000001425), *Thermoplasma acidophilum* (UP000001024), *Thermotoga maritima* (UP000008183), *Vibrio cholerae*

(UP000000584), and *Yersinia pestis*(UP000000815). These files were used to compute IDR boundaries with IUPred2A with values above 0.5 considered disordered. The resultant IDRs were then projected to the associated AlphaFold structural models as obtained from the AFDB.

Disease mutations

The missense variants from the OMIM database (35) annotated in UniProtKB were available for download on the UniProt website (humsavar.txt, September 2019). An in-house Python script was used to map the OMIM missense mutations to SPOT-Disorder predicted IDRs that are greater than or equal to 10 residues in length. We next assessed the differences in per-residue missense mutation rates in IDRs with different pLDDT scores, as annotated in the AFDB. Specifically, we focused on the difference in the per-residue mutation rates in IDRs with very high (≥ 90), high (≥ 70), or low (< 50) pLDDT scores. As a control, we also assessed the per-residue mutation rates of presumably non-pathogenic variants from the 1000 Genome Project (1000GP) (36), which were annotated in UniProtKB and available for download from the UniProt website (homo_sapiens_variation.txt, September 2019). As above, we mapped the missense variants to IDRs that were greater than or equal to 10 residues in length, and we then split the IDRs into three categories based on pLDDT scores. The Fisher Exact Test was used to assess the significance of the difference in per-residue mutation rates in regions with very high and high pLDDT scores as compared to regions with low pLDDT scores. In all four comparisons (OMIM: very high vs. low, high vs. low; 1000GP: very high vs. low, high vs. low), the p value was less than 0.0001.

FoldX

The AlphaFold2 structural model of ALX3 was downloaded from the AFDB. Residues corresponding to the homeodomain (152-213) were excised and saved as a separate PDB file. The homeodomain PDB file was then supplied as input for the FoldX command “RepairPDB”. Version 4 of FoldX was used (37). The mutation L168V was introduced into the output PDB file by the FoldX command “BuildModel”, with the number of runs set to five. The average ΔΔG value and standard deviation were computed over these five runs. The FoldX protocol was based on (38). Inter-atomic interactions in the wild-type and mutant PDB files

were analyzed with the Arpeggio webserver (39). Cavity detection was performed in PyMol with a cavity detection radius and cavity detection cutoff both set to 3 solvent radii.

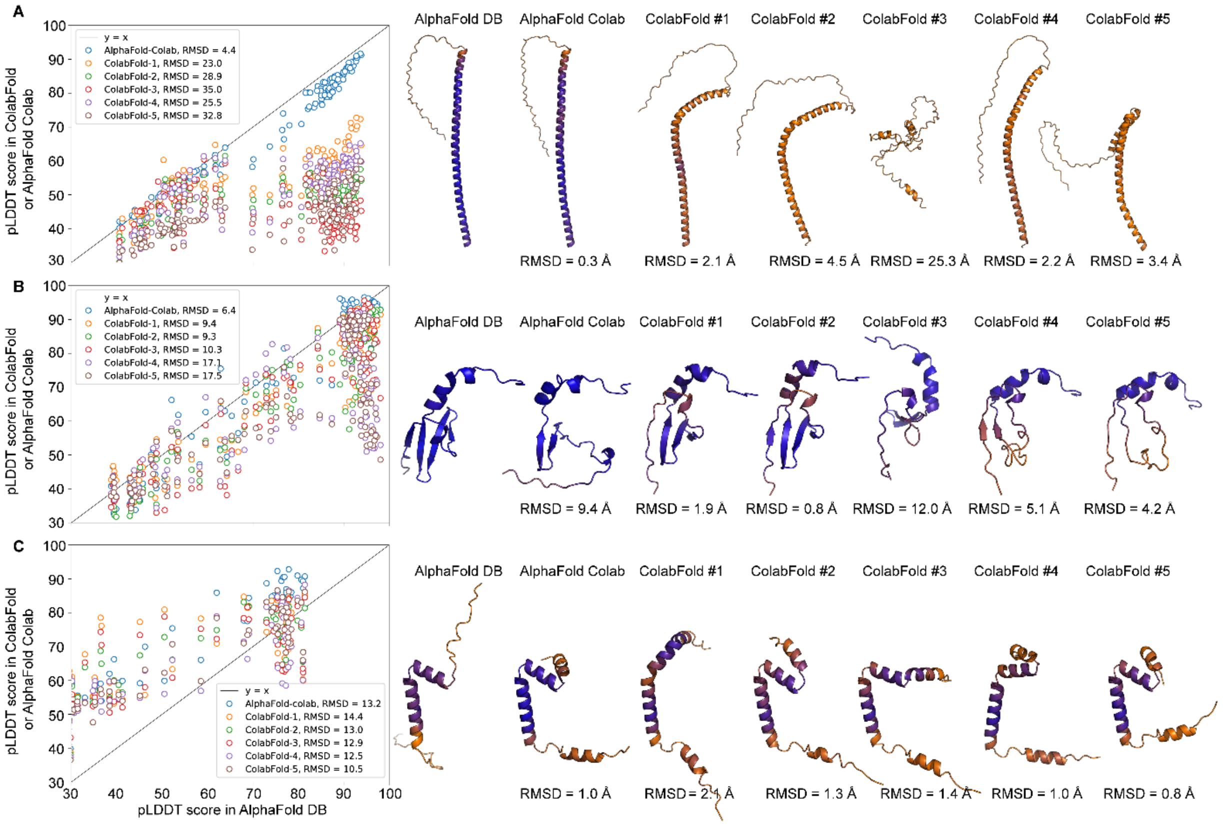

Supplementary Figure 1. Comparison of the structural predictions by the AFDB and other online versions of AlphaFold2. ColabFold (40) and the AlphaFold Colab notebooks were used to generate structural predictions of human α-synuclein (UniProt: P37840), 4E-BP2 (Q13542), and ACTR (Q9Y6Q9; residues 1123-1193). The per-residue pLDDT scores were extracted from the resultant structure (AlphaFold Colab) or the five models produced by ColabFold and compared to those within the AFDB structure. The root-mean-squared-deviation (RMSD) of the pLDDT scores is listed for the comparison of AFDB with AlphaFold Colab or ColabFold. *Right*: the structures generated from the AFDB, AlphaFold Colab, and ColabFold calculations. Note that AlphaFold Colab returns only one model whereas ColabFold returns five models. The RMSD values over regions of secondary structure upon alignment to the AFDB model are listed.

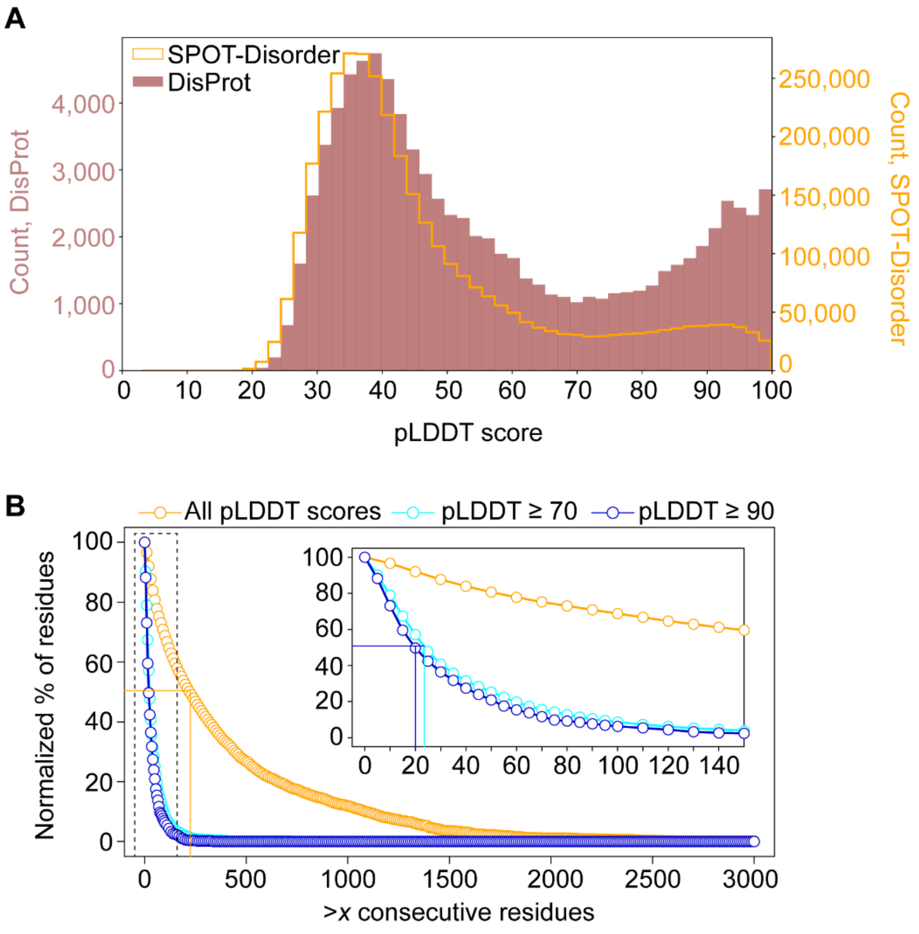

Supplementary Figure 2. **Filtering the human AFDB with DisProt and for regions of consecutive disorder.** (**A**) Histogram of per-residue pLDDT scores for proteins in the human AFDB filtered by SPOT-Disorder-predicted regions of intrinsic disorder (orange) or experimentally validated IDRs from DisProt (maroon). The percentage of residues with pLDDT scores ≥ 70 is 14.3% for SPOT-Disorder and 29.5% for DisProt. (**B**) SPOT-Disorder-predicted disordered residues (orange) were filtered for regions that contained greater than *x* consecutive residues that were disordered. Long disordered regions are abundant in the human proteome, with over 50% of SPOT-Disorder-predicted disordered residues falling in regions that have more than 220 consecutive residues (orange line). When only SPOT-Disorder-predicted IDRs that have pLDDT scores ≥ 70 (cyan) or ≥ 90 (blue) are analyzed, the percentage of residues that have long, consecutive regions of disorder is dramatically smaller, with 50% of residues in these groups of IDRs having more than 24 (cyan line) or 20 (blue line) consecutive residues for pLDDT scores ≥ 70 and ≥ 90, respectively. The y-axis shows the normalized percentage of residues, *i.e.* the sum of the consecutive residues for each threshold divided by the total number of residues in each group of IDRs.

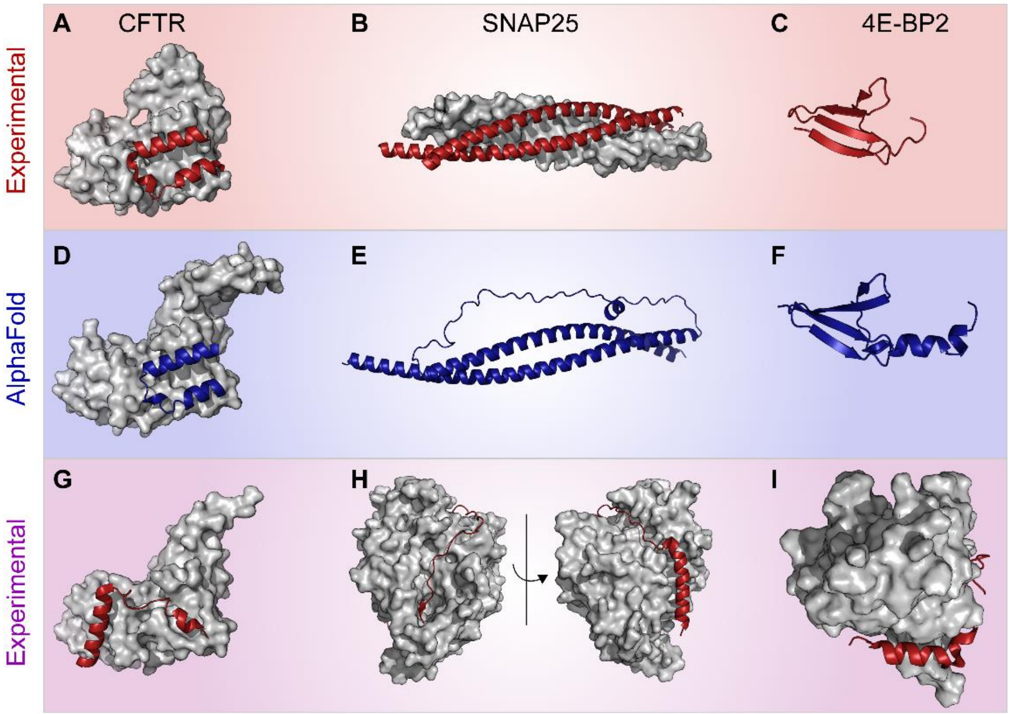

Supplementary Figure 3. **The AFDB does not capture the inherent structural plasticity of IDRs/IDPs.** Three examples of IDRs/IDPs that have experimentally determined structures when bound to interacting partners (intra- or intermolecular). (**A**) The disordered regulatory extension (RE) of human CFTR (residues L636-S670) bound intramolecularly to NBD1 (residues S388-S635; PDB ID: 1r0x). Note that the N-terminal region of NBD1 contains a cloning artifact and so residues S388-I393 differ from the AlphaFold2 model. (**B**) The N- and C-terminal SNARE motifs of human SNAP25 (residues S10-L81 and G139-W204, respectively) bound to rat VAMP2 (residues S28-N93), rat syntaxin-1A (residues L192-D250), and rat complexin-1 (residues K32-I72; PDB ID: 1kil). (**C**) Phosphorylated 4E-BP2 (residues P18-R62; PDB ID: 2mx4. (**D**-**F**) The AlphaFold2-predicted structures (blue) of the CFTR RE (residues P638-S670 in blue), SNAP25 (M1-G206), and 4E-BP2 (A16-P72) show excellent correspondence with the experimental structures in A-C. Note that the full-length protein sequences were used for structure predictions. (**G**-**I**) However, the CFTR RE, SNAP25, and 4E-BP2 have also been captured in a different conformation as those in panels A-C (red). (**G**) CFTR RE (P638-L671) intramolecularly bound in a different orientation to NBD1 (S388-Q637; PDB ID: 1xmi). (**H**) SNAP25 C-terminal SNARE motif (residues M146-G204) bound to BoNT/A) protease (residues P2-R425; PDB ID: 1xtg). (**I**) A peptide from 4E-BP2 (residues T50-P84) bound to eIF4E (residues H33-K206; PDB ID: 5bxv).

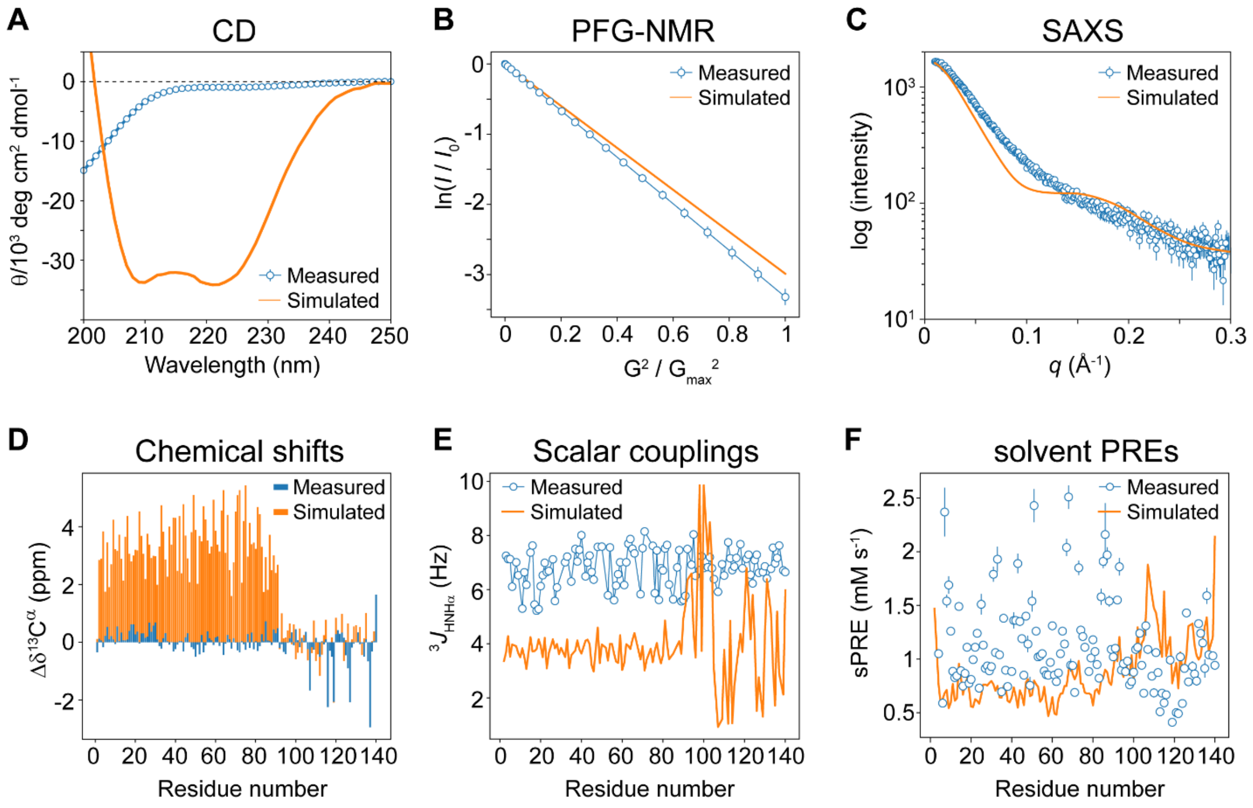

Supplementary Figure 4. **Integrative biophysical approach to evaluate high-confidence structures of IDRs in the AFDB**. Biophysical experiments can rapidly determine if an AFDB structure accurately captures the global properties of the protein in solution. Experimental (**A**) CD spectrum (24), (**B**) PFG-NMR translational diffusion (41), and (**C**) small-angle X-ray scattering (SAXS) data (28) for α-synuclein are shown in blue. Simulated data based on the AFDB structure of α-synuclein are shown in orange. These experiments require a limited amount of unmodified, natural abundance protein samples. If NMR assignments are available, local structural details can be compared, although such experiments require considerably more time as well as isotope-enriched samples. Experimental NMR-derived data for α-synuclein are shown in blue: (**D**) secondary ^{13}Cα chemical shifts (3), (**E**) $^{3}J_{HNH\alpha}$ coupling constants (42), and (**F**) ^{1}Hα solvent paramagnetic relaxation enhancements (PREs) (33). Even if NMR assignments are not available, the data shown in **D**-**F** can be plotted as histograms to compare experimental results with the simulations. The secondary ^{13}Cα shifts can be recomputed as ^{13}Cα – ^{13}Cβ shifts, because the secondary shifts require knowledge of the random coil shifts, which would not be known without assignments. See the Supplementary Appendix for more details.

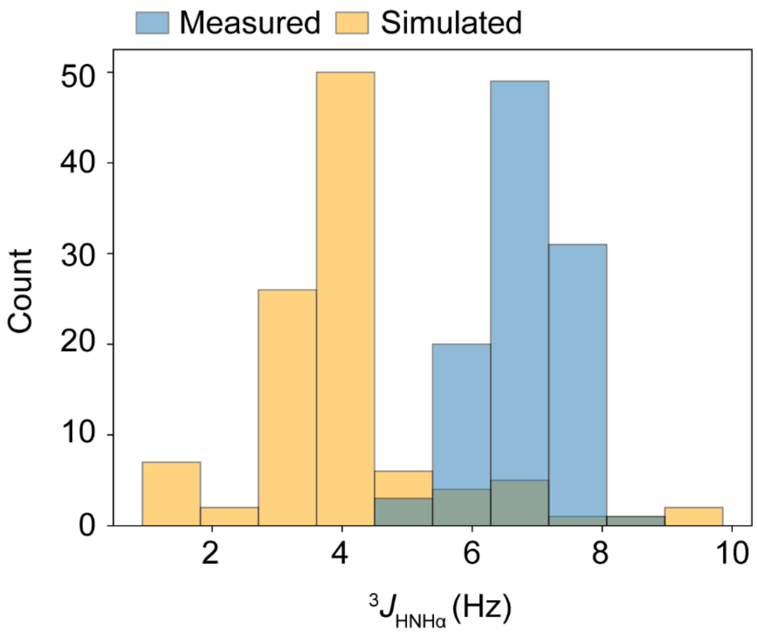

Supplementary Figure 5. **Using NMR data without chemical shift assignments to assess an AlphaFold2 prediction**. $^{3}J_{HNH\alpha}$ coupling constants (42) from α-synuclein (blue) are shown in a histogram format. The histogram of $^{3}J_{HNH\alpha}$ values that are back-calculated from the AlphaFold2 structural model are shown in orange. This histogram approach for NMR values mimics a situation in which resonance assignments are not available. A comparison between the histograms shows clear discrepancies between the experiment and simulation, even when no assignments are available.

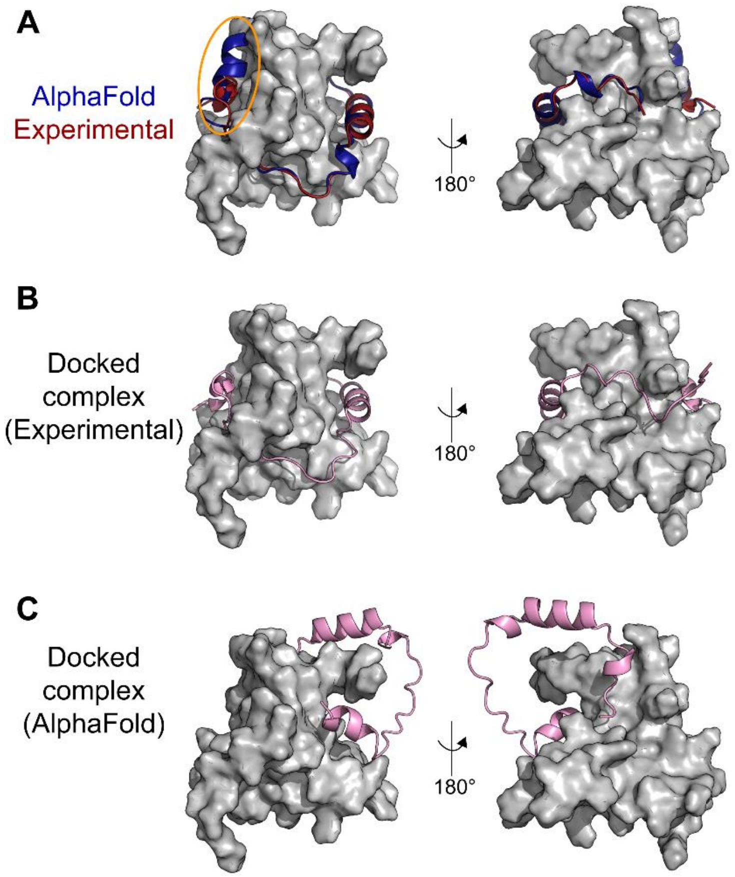

Supplementary Figure 6. The structures of IDRs in the AFDB, even if confident, may yield incorrect results when used to obtain a structural model of a protein complex using molecular docking. (**A**) Overlaid structures of the CITED2 TAD bound to CBP TAZ1 that was experimentally determined (red, PDB 1p4q) and the AlphaFold-predicted structure of the CITED2 TAD (blue). The TAZ1 domain is shown in a grey surface representation and was not included in the AlphaFold prediction of the structure for the CITED2 TAD. The orange ellipse indicates the position of the CITED2 C-terminal helix whose orientation differs in the AlphaFold2 model with respect to the experimental structure. The lowest-energy model returned by FRODOCK2.0 (43) when docking the CITED2 TAD with the (**B**) experimentally determined structure or the (**C**) AlphaFold-predicted structure onto the CBP TAZ1. Note that the C-terminal helix of CITED2 does not interact with the correct binding pocket in the CBP TAZ1 domain when using the AlphaFold2 model for docking purposes. See text for residue boundaries.

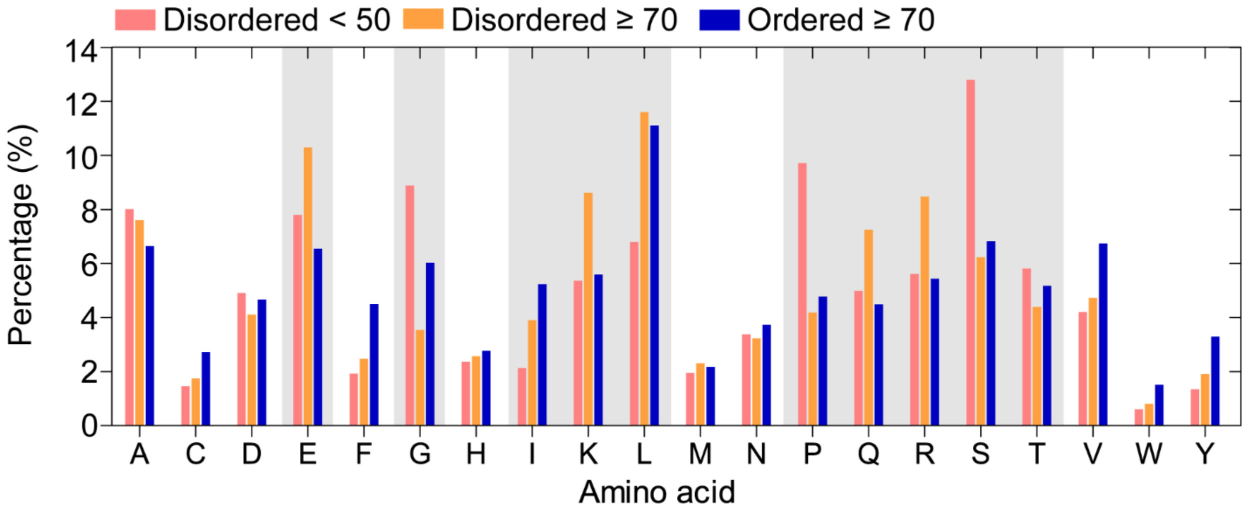

Supplementary Figure 7. Amino-acid frequencies in the human AFDB as a function of pLDDT score. The human AFDB was filtered with SPOT-Disorder-predicted regions of disorder to obtain predicted disordered and ordered regions. Plotted here are amino-acid percentages (eq 1, Methods) in predicted disordered regions with pLDDT scores < 50 (salmon), predicted disordered regions with pLDDT scores ≥ 70 (orange), or predicted ordered regions (blue). Amino acids with particularly significant differences between the two disordered datasets are indicated with grey boxes. These raw percentages were used to compute the amino acid differences shown in Figure 4.

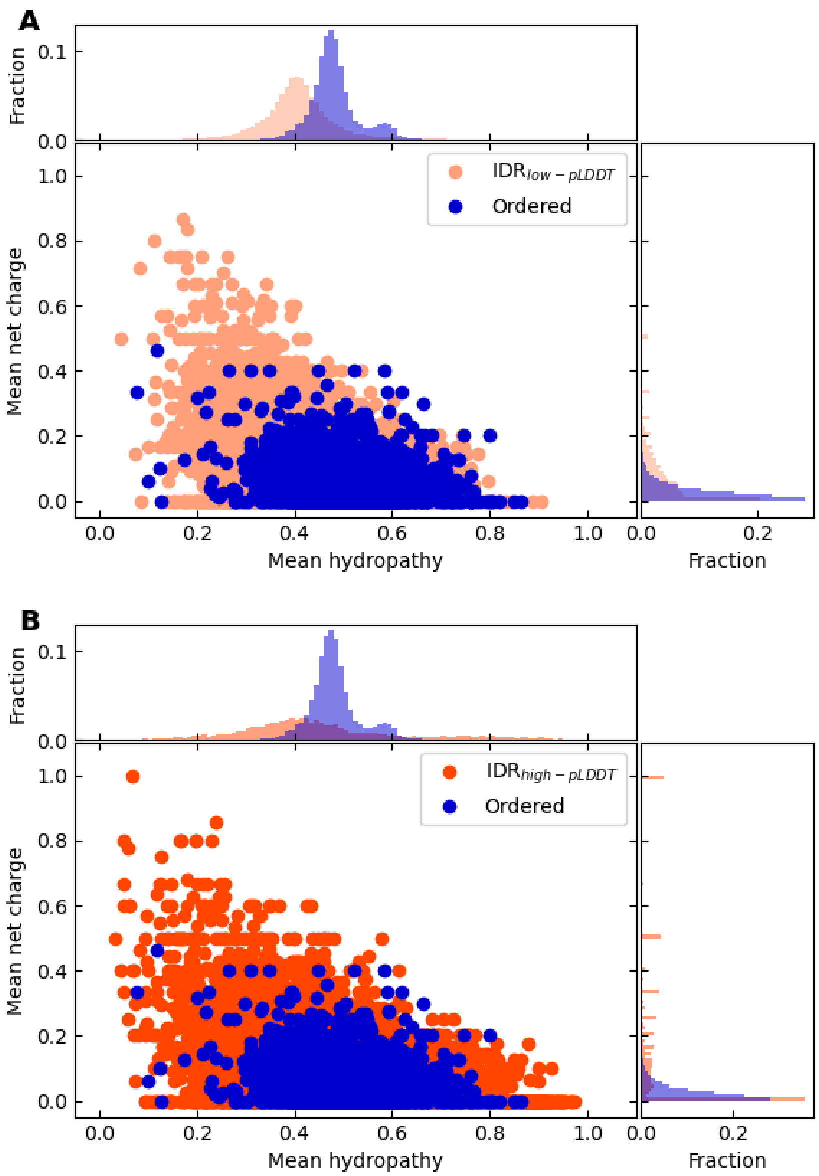

Supplementary Figure 8. Predicted IDRs in the human AFDB with high-confidence structures resemble IDR sequences. Analysis of the mean net charge and mean hydropathy values of predicted IDRs that have very low (≤ 50, $IDR_{low\text{-}pLDDT}$) (**A**) or very confident (**B**) (≥ 90, $IDR_{high\text{-}pLDDT}$) pLDDT scores as compared to ordered regions. The histograms are normalized such that the sum of all values equals unity.

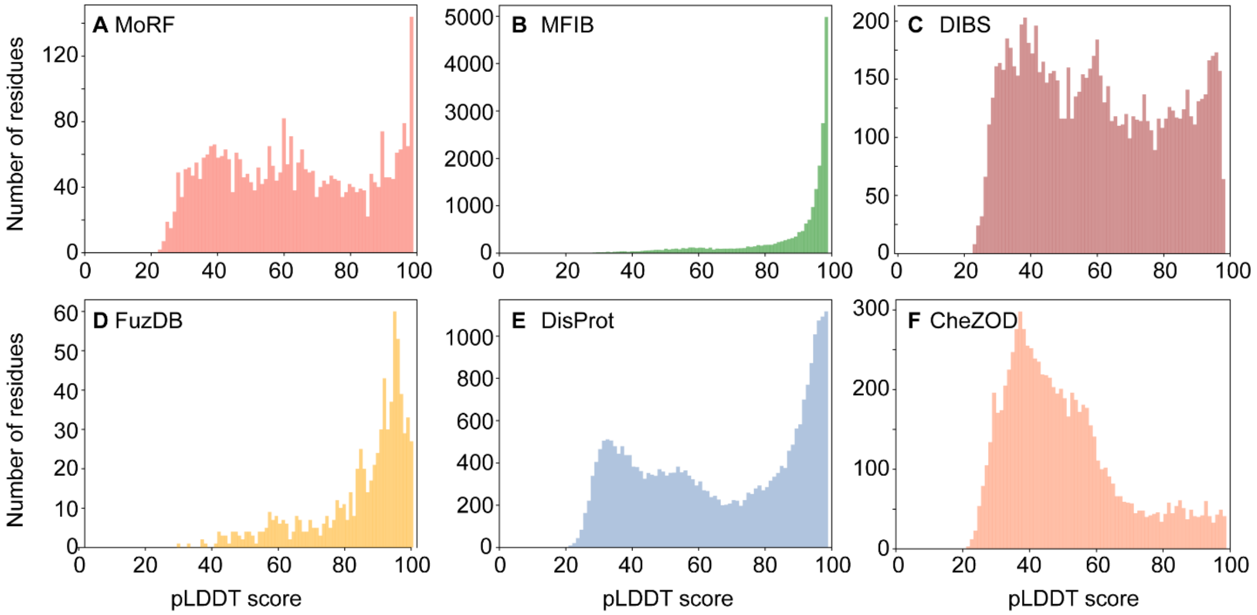

Supplementary Figure 9. Per-residue pLDDT scores for IDRs that were used to classify conditional folders. Analysis of per-residue pLDDT scores for known IDRs/IDPs that fold upon binding or modification. These examples were taken from the following five databases: MoRF (**A**), MFIB (**B**), DIBS (**C**), FuzDB (**D**), and DisProt (**E**). Of the IDR sequences in these databases, only the regions that map to the corresponding predicted structure in the AFDB were retained for this analysis. See main text for details. The y-axis shows the counts for the pLDDT scores of the IDRs/IDPs in the indicated databases. Panels **A** – **E** were used as true positive datasets in the ROC analysis. For comparison, the pLDDT score distribution from the true negative dataset (CheZOD, filtered as described in the text), is shown in (**F**). The IDRs in the filtered CheZOD dataset have not been reported to conditionally fold, nor are they found in the PDB with coordinates. Note that the pLDDT score distribution for these IDRs is shifted toward lower values.

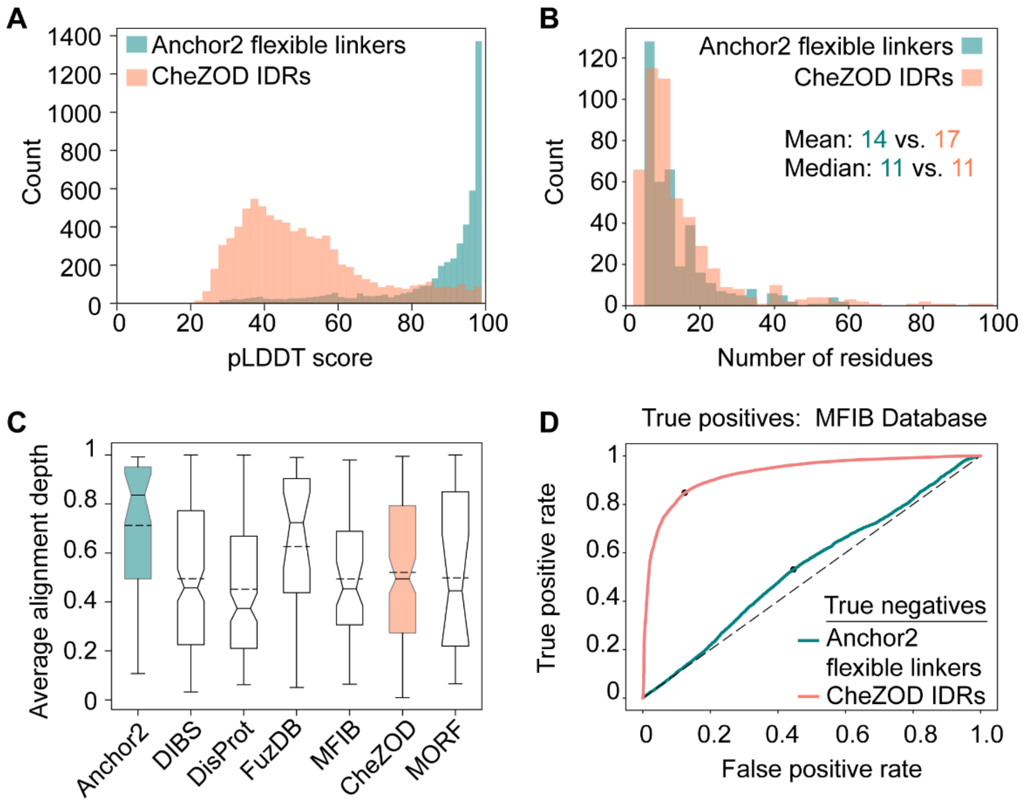

Supplementary Figure 10. **Comparison of NMR-validated IDRs from CheZOD with the dataset of flexible linkers used by Anchor2.** (**A**) Distribution of per-residue pLDDT scores for the IDR sequences in the Anchor2 true negative dataset of flexible linkers (blue). Per-residue pLDDT scores for the CheZOD-filtered set of IDRs that do not conditionally fold are shown in orange. The distribution of pLDDT scores for the filtered CheZOD dataset more closely resembles IDRs in the human proteome (Figure 1). (**B**) The distribution of IDR sequence lengths in the Anchor2 flexible linkers dataset (blue) and the CheZOD-filtered IDR dataset (orange). The mean and median values for each dataset are indicated (Anchor2 vs. CheZOD). (**C**) Box plots showing the average alignment depth for the IDR sequences in the Anchor2 flexible linker dataset (blue) as compared to the filtered CheZOD dataset (orange). For comparison, the average alignment depths are also shown for the five true positive databases of IDRs that are known to conditionally fold (DIBS, DisProt, FuzDB, MFIB, MORF). The IDRs in the flexible linkers dataset from Anchor2 have the deepest sequence alignments. (**D**) ROC curves for the binary classification task of identifying conditionally folded IDRs based on pLDDT scores alone. The true positive dataset was the MFIB database and the true negatives were either the Anchor2 flexible linkers (blue) or the filtered set of CheZOD IDRs (orange). The classification performance greatly increases when the CheZOD-derived IDRs are used as a set of true negatives.

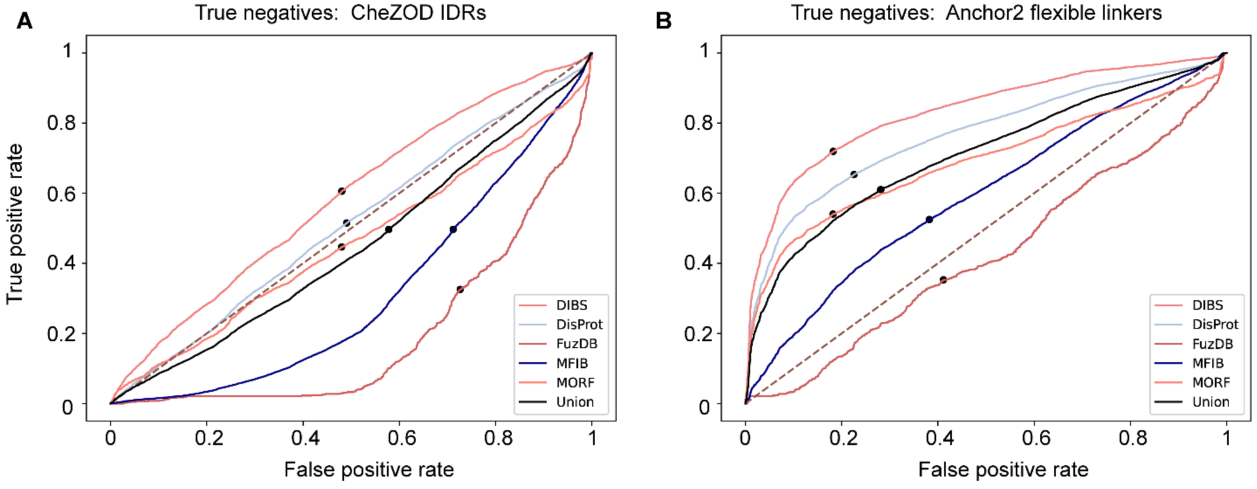

Supplementary Figure 11. Performance of the Anchor2 software as a classifier of conditionally folding IDRs. (**A**) Anchor2 was run with default parameters using the sequences of IDRs from five databases of conditionally folded IDRs (MFIB, DIBS, DisProt, FuzDB, MoRF) as input. The set of true negatives was the filtered set of NMR-validated IDRs in CheZOD (see Supplementary Figure 8). An ROC analysis was performed on these data. Anchor2 performs well at classifying the IDRs within the DIBS dataset, although some of these IDRs were used in the training of the Anchor2 software. The ROC curves when using AlphaFold2 pLDDT scores as input data are shown in Figure 4A for comparison. (**B**) The same analysis but with the flexible linkers dataset from supplied as true negatives (see text, Supplementary Figure 10), which was used for the training and testing of Anchor2. The AUC for DIBS is 0.83 and closely recapitulates the original Anchor2 performance (20). The black dot on each curve in (A) and (B) represents the threshold at which the true-to-false positive ratio is maximized.

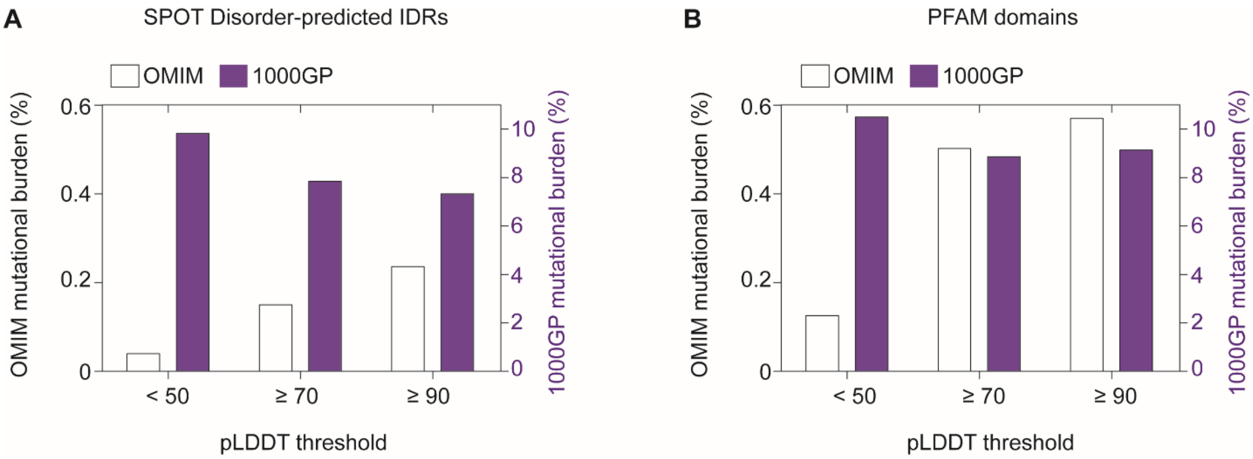

Supplementary Figure 12. Mapping of pathogenic and non-pathogenic mutations to SPOT-Disorder-predicted IDRs and PFAM domains. As described in the text, pathogenic mutations were obtained from OMIM and presumably non-pathogenic mutations from the 1000GP dataset. The mutations were then mapped to protein-coding genes in the human proteome and further filtered into SPOT-Disorder-predicted IDRs (**A**) or PFAM domains that were filtered to remove any overlap with the former (**B**). The mutational burden (%) is calculated as the total number of mutations divided by the total number of residues considered. Panels **A** and **B** are shown as a function of the pLDDT score threshold, with OMIM mutations shown in white bars (left y-axis) and 1000GP mutations in purple bars (right y-axis). Note that panel **A** is the same data that are plotted in **Figure 5A** of the main text; this graph is included here to enable a direct comparison to the PFAM domains. The number of mutations that map to each of the different pLDDT thresholds for both of the mutational datasets is listed in the main text.

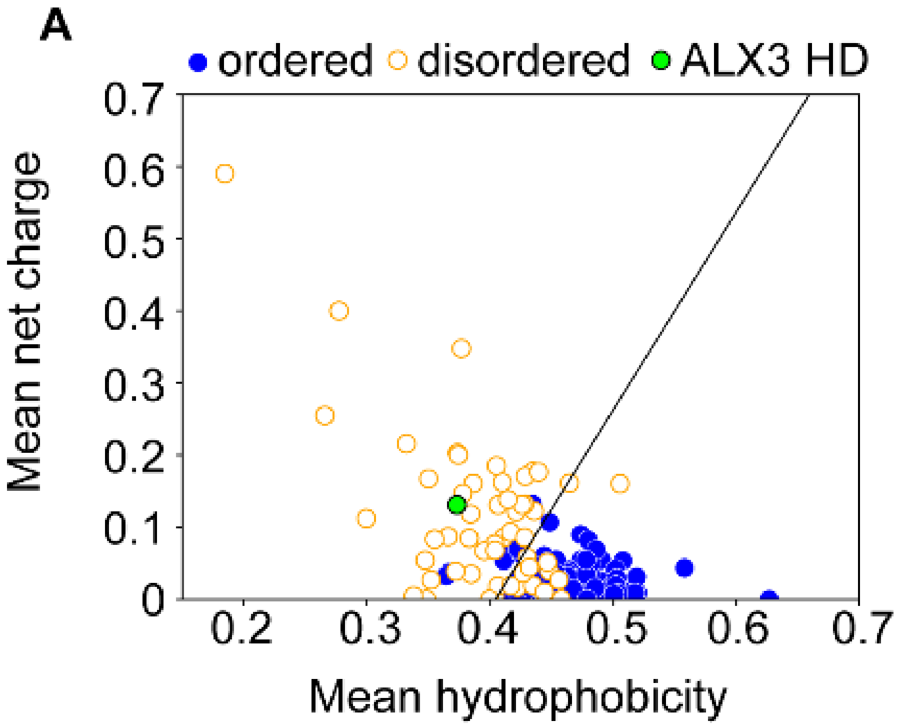

Supplementary Figure 13. Sequence analysis of the ALX3 homeodomain. (**A**) Uversky plot showing ordered (blue) and disordered (orange) proteins separated by the mean hydrophobicity and mean net charge of the sequence. The ALX3 homeodomain (green), residues 153-213, resembles the sequence properties of disordered proteins. The ordered and disordered proteins were extracted from the PONDR website (44).

Supplementary Table 1. **Segmenting the AFDB into predicted regions of disorder and order**. SPOT-Disorder and IUPred2A were used to predict IDRs within the human proteome and then to segment the AFDB into ordered and disordered regions. The number of residues that are not mapped from the human proteome to the AFDB are listed in the final column. The percentage of the predicted ordered, predicted disordered, and not mapped residues are listed in parentheses.

Dataset used to filter AFDB	Total residues	Ordered (%)	Disordered (%)	Not mapped (%)
SPOT-Disorder	10,825,508	7,127,685 (65.84%)	3,539,799 (32.70%)	158,024 (1.46%)
IUPred2A	10,825,508	7,942,769 (73.37%)	2,714,596 (25.08%)	168,143 (1.55%)

Supplementary Table 2. Per-residue pLDDT scores for predicted disordered and ordered regions of the human proteome. SPOT-Disorder was used to predict disordered regions of the human proteome and then to segment the human AFDB into regions of predicted disorder and order. The percentage of residues are listed as a function of the per-residue pLDDT score. Values listed in parentheses were obtained when IUPred2A was used to predict regions of disorder.

Dataset	*N* residues	% pLDDT ≥ 90	% 90 > pLDDT ≥ 70	% 70 > pLDDT ≥ 50	% pLDDT < 50
Proteome	10,825,508	38.12	24.46	9.85	27.57
Ordered	7,127,685	54.51	31.82	7.46	6.21
Disordered	3,539,799	4.52 (7.39)	9.78% (10.36)	14.73 (12.88)	70.98 (69.37)

Supplementary Table 3. SPOT-Disorder-predicted IDRs in the human proteome that have high- or very high-confidence AlphaFold2 pLDDT scores. SPOT-Disordered-predicted IDRs in the human proteome of a minimum length of 10 or 30 or more consecutive residues are quantified. The pLDDT score is also considered, with any pLDDT score (*all*) or only residues with pLDDT scores greater than or equal to 70 or 90. *N* IDRs refers to the number of regions, *N* proteins refers to the number of unique proteins (*i.e.*, multiple IDRs may come from a single protein), and *N* residues to the total number of amino acids. The superscripts [a] and [b] indicate the number of residues in each pLDDT threshold divided by the total number of disordered residues for a length cut-off of ≥ 10 and ≥ 30, respectively.

pLDDT score	Length cut-off	*N* proteins	*N* IDRs	*N* residues
all	≥ 10 AA	17,333	37,616	3,398,344
≥ 70	≥ 10 AA	7,586	14,996	400,244 (11.8%)[a]
≥ 90	≥ 10 AA	2,981	4,883	121,314 (3.6%)[a]
all	≥ 30 AA	13,070	20,492	3,097,338
≥ 70	≥ 30 AA	2,409	3,730	210,584 (6.8%)[b]
≥ 90	≥ 30 AA	843	1,157	61,745 (2%)[b]

Supplementary Table 4. Secondary structure content in the predicted disordered and ordered regions as a function of the pLDDT score. SPOT-Disorder was used to segment the human AFDB into predicted regions of disorder and order. DSSP was used to assign secondary structure.

Dataset	pLDDT score	Helix (%)	Coil (%)	Strand (%)
Ordered	≥ 90	47.2%	28.5%	24.3%
	90 > *x* ≥ 70	44.0%	42.6%	13.4%
	70 > *x* ≥ 50	33.2%	61.7%	5.1%
	< 50	10.5%	87.8%	1.7%
Disordered	≥ 90	78.1%	16.1%	5.8%
	90 > *x* ≥ 70	66.5%	29.0%	4.5%
	70 > *x* ≥ 50	30.8%	67.4%	1.8%
	< 50	3.4%	96.4%	0.2%

Supplementary Table 5. Classification performance statistics from the ROC analysis of conditionally folded IDRs. Listed here are the AUC, recall, and specificity values from the ROC analysis of AlphaFold2 per-residue pLDDT scores in classifying conditionally folded IDRs. The true positive databases are listed under "Database name" and the set of true negatives was the filtered CheZOD list.

Database name	AUC	RECALL	SPECIFICITY
MFIB	0.93	0.85	0.88
FuzDB	0.90	0.83	0.84
DisProt	0.68	0.56	0.77
MoRF	0.67	0.55	0.75
DIBS	0.63	0.53	0.70
Union (all)	0.76	0.63	0.82

Supplementary Appendix

Structural plasticity of IDRs

Here, we discuss the three examples of IDRs with multiple experimental structures shown in Figure 4 at length. The regulatory (R) region of CFTR is a long IDR that is heavily phosphorylated with several regions that adopt residual helical propensity (45, 46). The weak, multivalent inter- and intramolecular interactions between the R region and different binding partners regulate the activity of CFTR in a phosphorylation-dependent manner (46). The AlphaFold2 model of the portion of the CFTR R region immediately following the first nucleotide-binding domain (NBD1), called the regulatory extension (RE), shows close agreement with its conformation in a crystal structure of NBD1 and the RE (**Supplementary Figure 3D**). However, another structure of NBD1 shows the RE interacting with a different interface on NBD1, with the orientation of RE with respect to NBD1 dramatically altered, despite almost no changes to the structure of NBD1 itself (47). The AFDB structure of CFTR contains only one of these conformational states for the RE.

Another example is provided by SNAP-25, which is an IDP that folds into a helical bundle in SNARE complexes (**Supplementary Figure 3B**) that have important functions in membrane fusion during synaptic vesicle exocytosis. The AlphaFold2 model of SNAP-25 correctly identifies the N- and C-terminal soluble N-ethylmaleimide-sensitive factor attachment protein receptor (SNARE) motifs that form a four-helix bundle in the ternary SNARE complex involving SNAP-25, synaptobrevin, and syntaxin (**Supplementary Figure 3E**). However, SNAP-25 is also a substrate for Clostridal neurotoxins (CNTs), which are zinc-dependent endopeptidases that cause the diseases tetanus and botulism by specifically cleaving SNARE proteins and impairing neuronal exocytosis (48). The crystal structure of the botulinum neurotoxin serotype A (BoNT/A) protease bound to SNAP-25 reveals an extensive interface involving the C-terminal SNARE motif of SNAP-25 (49) (**Supplementary Figure 3I**). An α-helix is formed in BoNT/A-bound SNAP25 by residues D147-M167, while the remaining residues G168-G204 of SNAP-25 are bound to BoNT/A in a coil conformation, with a small β-strand (K201-L203) formed as well (49) (**Supplementary Figure 3I**). By contrast, in the SNARE complex bound to complexin-1, SNAP25 forms a long α-helix that encompasses residues S140-M202 (50) (**Supplementary Figure 3B**). These three structures of SNAP-25 provide an illustrative example: given the very high confidence in the AlphaFold2 structure of SNAP-25, one could assume that an ordered-to-disordered transition is required for SNAP-25 to bind to BoNT/A in the experimentally observed conformation, and that SNAP-25 assembles into SNARE complexes as a rigid body with minimal structural changes. Both of these assumptions are in stark contrast to the known disordered-to-ordered transition that occurs both upon binding to BoNT/A and formation of the SNARE complex. Thus, the molecular mechanism of SNAP-25 function, and its proteolytic cleavage in disease, are obscured by the high-confidence AlphaFold2 model.

Finally, we examined the case of 4E-BP2, an IDP that is a regulatory binding protein for the eIF4E, with experimental structures of segments of the protein in the 5-site phosphorylated state and in the non-phosphorylated state bound to eIF4E (**Supplementary Figure 3C, 3F, 3J**), as discussed in the section above. The AlphaFold2 structure of residues A16-P72 of 4E-BP2 contains a β-sheet followed by α- and 3_{10}-helices (**Supplementary Figure 3F**), whereas the experimental structure in phosphorylated 4E-BP2 contains the β-sheet for residues T19-D55 followed by a coil region (6) (**Supplementary Figure 3C**). The AlphaFold2 model correctly places the β-strands in phosphorylated 4E-BP2 and accurately identifies the orientations of each strand relative to one another (**Supplementary Figure 3F**). However, the helical secondary structure elements are only observed when non-phosphorylated 4E-BP2 binds to eIF4E (PDB ID: 3am7) (**Supplementary Figure 3J**). Upon binding to eIF4E, a truncated peptide from 4E-BP2 was shown to fold into an α-helix between residues D55-D61 (**Supplementary Figure 3J**). The related protein 4E-BP1, for which more complete structural information is available when bound to eIF4E (51) (PDB ID: 5bxv), forms an α-helix between residues D55-R62 followed by a short turn and a 3_{10}-helix between residues P66-Q69. The phosphorylation-induced folded state inhibits the binding of eIF4E that is otherwise extremely tight for the unmodified protein (6). The β-strand-rich structure of the AlphaFold2 model of 4E-BP2 is incompatible with the binding to eIF4E, which requires the disordered non-phosphorylated state known to fractionally sample helical structure in the segment that forms a stable α-helix in complex with eIF4E (5). Thus, the 4E-BP2 AFDB structure reflects a strange mixture of the ordered landscape of the protein, combining that found in the presence of PTMs with that stabilized in the absence of PTMs but in

the presence of a protein binding partner, and confounding understanding of the mechanism of phospho-regulation of translation initiation (6).

Rapid experimental assessment of the accuracy of AlphaFold2 predictions

In order to rapidly determine if the AlphaFold structure of an IDR/IDP is accurate, it is necessary to collect biophysical data on a purified sample of the IDR/IDP of interest. While biophysical experiments that can be performed inside living cells or in cell lysate may also be applicable (52, 53), and do not require sample purification, we focus here on *in vitro* measurements performed on purified proteins as such experiments are performed more routinely. Moreover, it would be beneficial to minimize the time and cost associated with sample preparation, experimental acquisition, and data analysis. Therefore, we have concentrated on biophysical assays that can be performed on (1) the wild-type amino-acid sequence of the protein, *i.e.* there should be no mutations required for covalent linkage of fluorescent tags or spin labels, and (2) natural abundance protein in a standard buffer, *i.e.* there should be no need for isotope enrichment or D_2O-based buffers that are common in small-angle neutron scattering (SANS), NMR, and EPR (54–56). As an example, we have compared biophysical data recorded on the protein α-synuclein, for which a number of biophysical measurements are available, with those that were simulated from the AFDB structure (**Supplementary Figure 4**, **Supplementary Figure 5**).

A global measurement of secondary structure content from circular dichroism (CD) or NMR spectroscopy may prove valuable to quickly distinguish between plausible models. In the latter case, neither resonance assignments nor isotope-enrichment with ^{15}N or ^{13}C are needed for global secondary structure analysis by NMR (57). However, the NMR approach requires relatively high protein concentrations, or long acquisition times in case of limited sample amounts, and a skilled user to analyze the data. For CD spectroscopy, sample requirements are minimal, data can be acquired in a short time, and one can quickly identify the secondary structure with software programs that fit the experimental spectrum (58). The experimental CD spectrum can readily be compared to simulated CD spectra obtained from an input three-dimensional structure via the webserver PDB2CD (59). For example, the predicted CD spectrum of α-synuclein based on the structure in the AFDB has diagnostic minima at 208 and 222 nm, characteristic of helical secondary structure, whereas the experimental spectrum shows a minimum near 200 nm, which is indicative of random coil conformations (**Supplementary Figure 4A**). These features show that the experimental data reflect a largely random or statistical coil conformation whereas the spectrum of the AFDB structure would be dominated by signals from α-helical conformations.

In cases where global secondary structure quantification is insufficient to determine if the AFDB model is accurate, such as when the AlphaFold structure of an IDR may only have a small percentage of secondary structure, measurements that are sensitive to the shape of the molecule may provide more information content. For example, it has already been demonstrated that IDRs with low pLDDT scores can be too expanded relative to experimental measurements (60). Thus, dynamic light scattering (DLS), small-angle X-ray scattering (SAXS), and pulsed-field gradient diffusion NMR spectroscopy (PFG-NMR), which are all highly sensitive to the shape of a molecule, could quickly identify such structures as erroneous. Moreover, all of these experiments can be performed on label-free, natural-abundance protein samples in conventional H_2O-based buffers. The hydrodynamic properties that are sampled in DLS and PFG-NMR experiments can be simulated from a known structure using the HYDROPRO software package (61), while SAXS curves can be simulated from a known structure with Crysol (27). We have chosen here to focus on PFG-NMR and SAXS, because DLS is more sensitive for larger particles and may require high protein concentrations for shorter IDRs in order to achieve sufficiently high signal-to-noise. By contrast, both PFG-NMR and SAXS data can be collected on relatively short IDRs at low protein concentrations. Moreover, because IDRs typically tumble independently of globular domains and yield sharp signals, NMR can monitor IDRs within very large particles even when the signals from the globular domain cannot be detected (62).

The simulated PFG-NMR and SAXS data for α-synuclein (**Supplementary Figure 4B, 4C**). The PFG-NMR data show that α-synuclein diffuses faster than the expectation based on the AFDB structure (**Supplementary Figure 4B**), and the SAXS data show deviations in the low *q* regions (**Supplementary Figure 4C**). The fitted values of the radius of gyration (R_g) and maximum distance (D_{max}) are 35.6 ± 0.2 Å

and 109 Å, whereas the back-calculated values from the AFDB structure are 42.6 Å and 152 Å, respectively. Thus, both PFG-NMR and SAXS measurements would indicate that the AFDB model is too extended relative to the conformation in solution.

If global measurements are insufficient to determine if the AFDB model is accurate, then more detailed local structural parameters can be obtained from NMR spectroscopy (57). Such experiments, however, generally require resonance assignment in order to yield atomic-level information. The process of resonance assignment typically requires the preparation of ^{13}C,^{15}N-labeled protein and the collection of multiple sets of 3D NMR spectra, which requires considerable more time than the above-mentioned experiments. Therefore, the following experiments can no longer be classified as "rapid"; however, the information content afforded by these experiments is very high.

For example, the deviation of the measured ^{13}Cα chemical shifts from random coil values (secondary chemical shift) is highly sensitive to α-helical conformations (**Supplementary Figure 4D**). For β-strands, ^{13}Cβ shifts are more sensitive. A comparison of secondary ^{13}Cα chemical shifts for α-synuclein and those back-calculated from the AFDB structure with SPARTA+ (9) reveals strong α-helical conformations in the AFDB model that are not present in the measured data. The residue-specificity afforded by NMR further pinpoints the structural deviations to the first *ca.* 90 residues of the AFDB model. In addition, three-bond scalar couplings report on the intervening dihedral angles. In particular, the readily measurable $^{3}J_{HNH\alpha}$ coupling reports on the dihedral angle Φ. For α-synuclein, the measured $^{3}J_{HNH\alpha}$ coupling constants (42) are considerably more uniform than those back-calculated from the AFDB model using the parametrized Karplus equation (Methods, equation 3) (30) (**Supplementary Figure 4E**). As above, the residue-specificity of these measurements enables a site-by-site comparison of the coupling constants. However, if assignments are not available, then histograms of the raw values from unassigned peaks can still be used to perform a comparison with the simulated values (**Supplementary Figure 5**). Finally, solvent paramagnetic relaxation enhancements (sPREs) provide site-specific structural restraints that report on solvent accessibility and local structural conformation. The measured ^{1}Hα sPREs (33) are considerably larger for the first *ca.* 90 residues of α-synuclein than those back-calculated from the AFDB structure with sPRE-calc (34) (**Supplementary Figure 4F**). Collectively, the NMR experiments point to localized structural differences in the first *ca.* 90 residues of the AFDB structure relative to the conformation sampled in solution. Additional NMR experiments, such as residual dipolar couplings (RDCs), PREs with site-directed spin labelling, and ^{15}N relaxation provide further orientational and distance restraints that can be compared to a structural model.

Thus, relatively easy biophysical experiments (**Supplementary Figure 4A-C**) can be performed on purified proteins *in vitro* to quickly assess the accuracy of AFDB predictions. These experiments do not require the introduction of any labels or mutations and can be performed in standard buffers that are compatible with conventional biophysical and biochemical assays. More time-consuming NMR experiments (**Supplementary Figure 4D-F**) can be performed to obtain site-specific information. If assignments are not available, however, then histogram-type analyses can still be performed to obtain structural insight (**Supplementary Figure 5**).

Rigid-body docking with confidently AlphaFold2-predicted IDP/IDR structures

The above examples collectively demonstrate how high-confidence AlphaFold2 structures of IDRs/IDPs, which can offer insight into various structures that are accessible to the IDR/IDP, may also obscure the molecular mechanisms of these disordered regions. Next, we investigated this problem from the other side: if high-confidence structures of IDRs/IDPs are capturing the bound/modified states of IDRs/IDPs, then can such structures be used with protein-protein docking software to obtain structural models of IDR/IDP complexes bound to globular domains? If so, then high-confidence AlphaFold2 structures of IDRs/IDPs could be used with the goal of identifying the interfaces of IDR/IDP-globular domain complexes.

Indeed, AlphaFold2 structural models have been used in large-scale molecular docking studies, but the specific case of conditionally folded IDRs/IDPs has not yet been explored. Thus, we tested if the AlphaFold2 models of IDRs with high pLDDT scores could be used for rigid-body molecular docking with putative binding partners. To test this, we used as a model system an experimentally determined complex structure

of a conditionally folded IDR, the CITED2 transactivation domain (TAD), bound to the folded CBP TAZ1 domain (63–65) (PDB: 1p4q), since this enables a comparison to the experimental structure of the complex. Indeed, as we showed above, the AlphaFold2-predicted structure of the CITED2 TAD closely resembles the CBP-bound form (**Figure 4C, 4G, 4K**), with a heavy-atom RMSD between the experimental and AlphaFold2 structures of only 1.6 Å for the entire region and 1.0 Å when aligning the helices only (**Figure 4K**, **Supplementary Figure 5A**). As a control, we first extracted the individual chains for the CITED2 TAD and the CBP TAZ1 in the experimental structure, and rigid-body docked these two chains with protein-protein docking software (**Supplementary Figure 6B**). Reassuringly, the lowest-energy docked structure agreed with the experimental complex (heavy-atom RMSD for residues N216-F259: 0.9 Å), indicating that this strategy could potentially work for AlphaFold2 structures that have atomic-level accuracy to the conditionally folded state of the IDR.

Next, we rigid-body docked the AlphaFold2 structure of the CITED2 TAD onto the experimentally determined structure of the TAZ1 globular domain (**Supplementary Figure 6C**). The results from this simple docking exercise are quite striking: even though the AlphaFold2 structure of the CITED2 TAD closely agrees with the experimental structure of CITED2 (1.6-Å RMSD for all residues or 1-Å RMSD for helices-only), the orientation of the C-terminal helix in the AFDB structure is shifted by 90° relative to the experimental structure (**Supplementary Figure 6A**). The rotation of the C-terminal helix in CITED2 causes a steric clash with the CBP TAZ1 domain that dramatically alters the lowest-energy structure of the docked complex (**Supplementary Figure 6C**), leading to a completely different docked complex. Thus, if one naively used the AlphaFold2 structure of the CITED2 TAD to dock this structure into its interacting globular domain, the resultant molecular model would be flawed. Although only one example is presented here, the conclusions from this molecular docking exercise agree with other protein-ligand docking studies that used AlphaFold2 structural models [cite, cite, cite], which generally find that AlphaFold2 models yield docked complexes with lower-scoring metrics than high-resolution experimental structures.

Supplementary References

1. Tunyasuvunakool K, et al. (2021) Highly accurate protein structure prediction for the human proteome. *Nature* 596(7873):590–596.

2. Ulrich EL, et al. (2008) BioMagResBank. *Nucleic Acids Res* 36(Database issue).

3. Bermel W, et al. (2006) Protonless NMR experiments for sequence-specific assignment of backbone nuclei in unfolded proteins. *J Am Chem Soc* 128(12):3918–3919.

4. Chandra S, Chen X, Rizo J, Jahn R, Südhof TC (2003) A broken alpha-helix in folded alpha-Synuclein. *J Biol Chem* 278(17):15313–15318.

5. Lukhele S, Bah A, Lin H, Sonenberg N, Forman-Kay JD (2013) Interaction of the eukaryotic initiation factor 4E with 4E-BP2 at a dynamic bipartite interface. *Structure* 21(12):2186–2196.

6. Bah A, et al. (2015) Folding of an intrinsically disordered protein by phosphorylation as a regulatory switch. *Nature* 519(7541):106–109.

7. Ebert MO, Bae SH, Dyson HJ, Wright PE (2008) NMR relaxation study of the complex formed between CBP and the activation domain of the nuclear hormone receptor coactivator ACTR. *Biochemistry* 47(5):1299–1308.

8. Demarest SJ, et al. (2002) Mutual synergistic folding in recruitment of CBP/p300 by p160 nuclear receptor coactivators. *Nature* 415(6871):549–553.

9. Shen Y, Bax A (2010) SPARTA+: a modest improvement in empirical NMR chemical shift prediction by means of an artificial neural network. *J Biomol NMR* 48(1):13–22.

10. Marsh JA, Singh VK, Jia Z, Forman-Kay JD (2006) Sensitivity of secondary structure propensities to sequence differences between alpha- and gamma-synuclein: implications for fibrillation. *Protein Sci* 15(12):2795–2804.

11. Maciejewski MW, et al. (2017) NMRbox: A Resource for Biomolecular NMR Computation. *Biophys J* 112(8):1529–1534.

12. Altschul SF, Gish W, Miller W, Myers EW, Lipman DJ (1990) Basic local alignment search tool. *J Mol Biol* 215(3):403–410.

13. Wang G, Dunbrack RL (2003) PISCES: a protein sequence culling server. *Bioinformatics* 19(12):1589–1591.

14. Capra JA, Singh M (2007) Predicting functionally important residues from sequence conservation. *Bioinformatics* 23(15):1875–1882.

15. Necci M, et al. (2021) Critical assessment of protein intrinsic disorder prediction. *Nat Methods* 18(5):472–481.

16. Uversky VN, Gillespie JR, Fink AL (2000) Why are “natively unfolded” proteins unstructured under physiologic conditions? *Proteins* 41(3):415–427.

17. Croke RL, Patil SM, Quevreaux J, Kendall DA, Alexandrescu AT (2011) NMR determination of pKa values in α-synuclein. *Protein Sci* 20(2):256–269.

18. Disfani FM, et al. (2012) MoRFpred, a computational tool for sequence-based prediction and characterization of short disorder-to-order transitioning binding regions in proteins. *Bioinformatics* 28(12):i75–i83.

19. Dosztányi Z, Mészáros B, Simon I (2009) ANCHOR: web server for predicting protein binding regions in disordered proteins. *Bioinformatics* 25(20):2745.

20. Mészáros B, Erdös G, Dosztányi Z (2018) IUPred2A: context-dependent prediction of protein disorder as a function of redox state and protein binding. *Nucleic Acids Res* 46(W1):W329–W337.

21. Nielsen JT, Mulder FAA (2020) Quantitative Protein Disorder Assessment Using NMR Chemical Shifts. *Methods Mol Biol* 2141:303–317.

22. Dass R, Mulder FAA, Nielsen JT (2020) ODiNPred: comprehensive prediction of protein order and disorder. *Sci Rep* 10(1): 14780.

23. Mavridis L, Janes RW (2017) PDB2CD: a web-based application for the generation of circular dichroism spectra from protein atomic coordinates. *Bioinformatics* 33(1):56–63.

24. Fusco G, et al. (2016) Structural basis of synaptic vesicle assembly promoted by α-synuclein. *Nat Commun 2016 71* 7(1):1–12.

25. Ortega A, Amorós D, García De La Torre J (2011) Prediction of Hydrodynamic and Other Solution Properties of Rigid Proteins from Atomic- and Residue-Level Models. *Biophys J* 101(4):892.

26. Ramanujam V, Alderson TR, Pritišanac I, Ying J, Bax A (2020) Protein structural changes characterized by high-pressure, pulsed field gradient diffusion NMR spectroscopy. *J Magn Reson* 312:106701.

27. Svergun D, Barberato C, Koch MH (1995) CRYSOL– a Program to Evaluate X-ray Solution Scattering of Biological Macromolecules from Atomic Coordinates. *J Appl Crystallogr* 28(6):768–773.

28. Ahmed MC, et al. (2021) Refinement of α-Synuclein Ensembles Against SAXS Data: Comparison of Force Fields and Methods. *Front Mol Biosci* 8: 654333.

29. Manalastas-Cantos K, et al. (2021) ATSAS 3.0: expanded functionality and new tools for small-angle scattering data analysis. *J Appl Crystallogr* 54(Pt 1):343–355.

30. Vögeli B, Ying J, Grishaev A, Bax A (2007) Limits on variations in protein backbone dynamics from precise measurements of scalar couplings. *J Am Chem Soc* 129(30):9377–9385.

31. Cock PJA, et al. (2009) Biopython: freely available Python tools for computational molecular biology and bioinformatics. *Bioinformatics* 25(11):1422–1423.

32. Mantsyzov AB, Shen Y, Lee JH, Hummer G, Bax A (2015) MERA: A webserver for evaluating backbone torsion angle distributions in dynamic and disordered proteins from NMR data. *J Biomol NMR* 63(1):85–95.

33. Hartlmüller C, Spreitzer E, Göbl C, Falsone F, Madl T (2019) NMR characterization of solvent accessibility and transient structure in intrinsically disordered proteins. *J Biomol*

NMR 73(6–7):305–317.

34. Gong Z, Gu XH, Guo DC, Wang J, Tang C (2017) Protein Structural Ensembles Visualized by Solvent Paramagnetic Relaxation Enhancement. *Angew Chemie* 56(4):1002–1006.

35. Amberger JS, Bocchini CA, Scott AF, Hamosh A (2019) OMIM.org: leveraging knowledge across phenotype-gene relationships. *Nucleic Acids Res* 47(D1):D1038–D1043.

36. Auton A, et al. (2015) A global reference for human genetic variation. *Nat 2015 5267571* 526(7571):68–74.

37. Schymkowitz J, et al. (2005) The FoldX web server: an online force field. *Nucleic Acids Res* 33(Web Server issue):W382.

38. Valanciute A, et al. (2022) Accurate protein stability predictions from homology models. *Comput Struct Biotechnol J* 21:66–73.

39. Jubb HC, et al. (2017) Arpeggio: A Web Server for Calculating and Visualising Interatomic Interactions in Protein Structures. *J Mol Biol* 429(3):365–371.

40. Mirdita M, et al. (2021) ColabFold - Making protein folding accessible to all. *bioRxiv*:2021.08.15.456425.

41. Ramanujam V, Alderson TR, Pritišanac I, Ying J, Bax A (2020) Protein structural changes characterized by high-pressure, pulsed field gradient diffusion NMR spectroscopy. *J Magn Reson* 312:106701.

42. Mantsyzov AB, et al. (2014) A maximum entropy approach to the study of residue-specific backbone angle distributions in α-synuclein, an intrinsically disordered protein. *Protein Sci* 23(9):1275–1290.

43. Ramírez-Aportela E, López-Blanco JR, Chacón P (2016) FRODOCK 2.0: fast protein-protein docking server. *Bioinformatics* 32(15):2386–2388.

44. Romero P, et al. (2001) Sequence Complexity of Disordered Protein. *Proteins* 42:38–48.

45. Baker JMR, et al. (2007) CFTR regulatory region interacts with NBD1 predominantly via multiple transient helices. *Nat Struct Mol Biol 2007 148* 14(8):738–745.

46. Bozoky Z, et al. (2013) Regulatory R region of the CFTR chloride channel is a dynamic integrator of phospho-dependent intra- and intermolecular interactions. *Proc Natl Acad Sci U S A* 110(47):E4427–E4436.

47. Bozoky Z, Krzeminski M, Chong PA, Forman-Kay JD (2013) Structural changes of CFTR R region upon phosphorylation: a plastic platform for intramolecular and intermolecular interactions. *FEBS J* 280(18):4407–4416.

48. Schiavo GG, et al. (1992) Tetanus and botulinum-B neurotoxins block neurotransmitter release by proteolytic cleavage of synaptobrevin. *Nature* 359(6398):832–835.

49. Breidenbach MA, Brunger AT (2004) Substrate recognition strategy for botulinum neurotoxin serotype A. *Nature* 432(7019):925–929.

50. Chen X, et al. (2002) Three-Dimensional Structure of the Complexin/SNARE Complex.

Neuron 33(3):397–409.

51. Sekiyama N, et al. (2015) Molecular mechanism of the dual activity of 4EGI-1: Dissociating eIF4G from eIF4E but stabilizing the binding of unphosphorylated 4E-BP1. *Proc Natl Acad Sci U S A* 112(30):E4036–E4045.

52. Gruebele M, Pielak GJ (2021) Dynamical spectroscopy and microscopy of proteins in cells. *Curr Opin Struct Biol* 70:1–7.

53. Gronenborn AM, Clore GM (1996) Rapid screening for structural integrity of expressed proteins by heteronuclear NMR spectroscopy. *Protein Sci* 5(1):174–177.

54. Gardner KH, Kay LE (1998) The use of 2H, 13C, 15N multidimensional NMR to study the structure and dynamics of proteins. *Annu Rev Biophys Biomol Struct* 27:357–406.

55. Schmidt T, Wälti MA, Baber JL, Hustedt EJ, Clore GM (2016) Long Distance Measurements up to 160 Å in the GroEL Tetradecamer Using Q-Band DEER EPR Spectroscopy. *Angew Chem Int Ed Engl* 55(51):15905–15909.

56. Clifton LA, et al. (2019) Structural Investigations of Protein-Lipid Complexes Using Neutron Scattering. *Methods Mol Biol* 2003:201–251.

57. Wishart DS, Sykes BD, Richards FM (1991) Simple techniques for the quantification of protein secondary structure by 1H NMR spectroscopy. *FEBS Lett* 293(1–2):72–80.

58. Greenfield NJ (2006) Using circular dichroism spectra to estimate protein secondary structure. *Nat Protoc* 1(6):2876–2890.

59. Mavridis L, Janes RW (2017) PDB2CD: a web-based application for the generation of circular dichroism spectra from protein atomic coordinates. *Bioinformatics* 33(1):56–63.

60. Ruff KM, Pappu R V. (2021) AlphaFold and Implications for Intrinsically Disordered Proteins. *J Mol Biol* 433(20):167208.

61. Ortega A, Amorós D, García De La Torre J (2011) Prediction of hydrodynamic and other solution properties of rigid proteins from atomic- and residue-level models. *Biophys J* 101(4):892–898.

62. Carver JA, Aquilina JA, Truscott RJW, Ralston GB (1992) Identification by 1H NMR spectroscopy of flexible C-terminal extensions in bovine lens alpha-crystallin. *FEBS Lett* 311(2):143–149.

63. De Guzman RN, Martinez-Yamout MA, Dyson HJ, Wright PE (2004) Interaction of the TAZ1 domain of the CREB-binding protein with the activation domain of CITED2: regulation by competition between intrinsically unstructured ligands for non-identical binding sites. *J Biol Chem* 279(4):3042–3049.

64. Freedman SJ, et al. (2003) Structural basis for negative regulation of hypoxia-inducible factor-1alpha by CITED2. *Nat Struct Biol* 10(7):504–512.

65. De Guzman RN, Wojciak JM, Martinez-Yamout MA, Dyson HJ, Wright PE (2005) CBP/p300 TAZ1 domain forms a structured scaffold for ligand binding. *Biochemistry* 44(2):490–497.
